# Microbial Community Profiling Distinguishes Left-Sided and Right-Sided Colon Cancer

**DOI:** 10.3389/fcimb.2020.498502

**Published:** 2020-11-26

**Authors:** Mengya Zhong, Yubo Xiong, Zhijian Ye, Jiabao Zhao, Lifeng Zhong, Yu Liu, Yuekun Zhu, Lantian Tian, Xingfeng Qiu, Xuehui Hong

**Affiliations:** ^1^ Department of Gastrointestinal Surgery, Zhongshan Hospital of Xiamen University, Xiamen, China; ^2^ School of Medicine, Xiamen University, Xiamen, China; ^3^ Department of General Surgery, The First Affiliated Hospital of Harbin Medical University, Harbin, China; ^4^ Department of Hepatopancreatobiliary Surgery of the Affiliated Hospital, Qingdao University, Qingdao, China

**Keywords:** morbidity, gut microbiota, left-sided colon cancer, right-sided colon cancer, targeted drug therapy

## Abstract

The difference between left- and right-sided colon cancer has become the focus of global attention, and researchers have found differences in the morbidity, molecular biological characteristics, and response to targeted drug therapy between left- and right-sided colon cancer. Therefore, the identification of more effective predictive indicators is critical for providing guidance to future clinical work. We collected samples from different colon sites and regions and analyzed the identities and distributions of differentially expressed species in the microbiota in the left and right sides of the colon to better explore the pathogenesis of colon cancer and provided a basis for individualized drug therapy. We collected samples from different regions in the body of 40 patients with colon cancer, including stool and tissues. The Subjects were classified into four groups, and this classification was mainly based on the colon cancer distribution. The microbiota composition of the left-sided and right-sided colon samples was assessed by specifically amplifying the V3-V4 region of the 16S rDNA gene from DNA extracts from the samples. These amplicons were examined by Illumina HiSeq 2500 sequencing. The microbial taxa in the left-sided colon samples are more abundant than those in the right-sided colon samples. The flora in the left-sided colon samples, such as *Clostridium perfringens* and *Fusobacterium nucleatum*, might be associated with VEGF expression and are more likely to promote colon cancer. The microbiota distribution in the right-sided colon samples is less invasive and harmful and particularly rich in *Bifidobacterium dentium*. In addition, *Streptococcus*, which is the target of EGFR, was found to be expressed in both the left- and right-sided colon samples but was found at a higher level in the left-sided colon samples. Additionally, the differential pathways involved in the left-sided colon samples mainly mediate DNA damage, methylation, and histone modifications, whereas those in the right-sided colon samples are dominated by DNA synthesis. The comparison of only the geographical differences revealed a significant difference in the distribution of the microbial population. The adherent microbiota composition and structural changes between the left- and right-sided colon samples might contribute to the development of colon cancer, lead to different morbidities, and further affect the prognosis of patients and their sensitivity to targeted drugs. Therefore, the identification of the differential flora in the colon could be used as an indicator for predicting the occurrence and development of colon cancer, which is also beneficial for future individualized drug therapy.

## Introduction

Colon cancer is a malignant tumor that seriously endangers human health (Wu et al., 2015). Although the quality of medical care is improving, the initial stage of the disease is relatively hidden, and the clinical manifestations lack specificity. Therefore, approximately 20 to 25% of patients are at an advanced stage of the disease at the time of diagnosis (Duffy et al., 2007). Colon cancer affects more than 250,000 people each year, and its late high mortality is one of the three leading causes of cancer-related deaths worldwide (Hattori et al., 2017). Bufill first proposed that colorectal cancer is a concept involving two different diseases (Bufill, 1990), and this hypothesis has gradually improved our understanding of the biological behavior of colon cancer. Researchers have attempted to identify more effective treatments based on different tumor characteristics. In the current era of individualized therapy for colon cancer, patients with colon cancer benefit greatly from individualized evaluation and proper medication.

Previous studies have found that the primary site of colon cancer is a potential factor affecting the pathogenesis and molecular characteristics of the disease (Missiaglia et al., 2014; Gao et al., 2017). Based on its different embryonic origins and the molecular and biological mechanisms of tumor formation in different regions of the large intestine, colon cancer has been divided into left-sided colon cancer (LCC) and right-sided colon cancer (RCC, found in the colonic spleen) (Boisen et al., 2013; Hussain et al., 2016). These two types of colon cancer exhibit significant differences in clinical features, morbidity, histology, molecular biology, targeted drug therapy, and prognosis, and thus, the treatment concepts for the two diseases are also different (Benedix et al., 2010a; Moritani et al., 2014; Lee et al., 2017). The available data show that LCC is more common than RCC, LCC has a higher incidence in males, and females are more susceptible to RCC. The average age of onset in RCC is significantly higher than that of LCC. With respect to targeted drug therapy, cetuximab, which targets the epidermal growth factor receptor (EGFR), is currently better for the treatment of LCC, whereas RCC patients exhibit a better response to treatment with the vascular endothelial growth factor (VEGF)-targeted drug bevacizumab (Benedix et al., 2010b; Boleij and Tjalsma, 2013; Li et al., 2017). In addition, the human large intestine is also one of the densest microbial ecosystems in the human body, and differences in the gut microbiota have become an important factor in determining the occurrence and prognosis of colon cancer (Wu et al., 2004; Camp et al., 2009). The human gastrointestinal tract is colonized by complex and diverse commensal microbial communities that contribute to the health of the host (Price et al., 2015; Garrett, 2019). The gut has approximately 40 trillion microbes, the vast majority of which are present in the large intestine (colon and rectum) (Sender et al., 2016; Wong and Yu, 2019; Taddese et al., 2020), and 60–80% of the microbes have not been identified due to culture-related difficulties (Van Citters and Lin, 2005; Shen et al., 2010). Therefore, the colon is the main contributor to the total number of bacteria in the digestive tract. The microbes in different regions of the colon of normal individuals are relatively uniform but whether the mucosal flora of patients with LCC and RCC exhibit differences remains vastly unclear (Flemer et al., 2017).

Previous studies have revealed that the prominent view of tumor type-specific intracellular bacteria is initially driven and triggered by the colonization of specific pathogens in the local mucosa, which subsequently results in changes in the surrounding environment of cancer and thereby allows the colonization of specific opportunistic pathogens, even though they are usually healthy flora in the intestine (Man et al., 2015; Mirzaei et al., 2020; Nejman et al., 2020). With the continuous introduction of individualized and precise treatments and due to the morbidity of LCC and RCC, VEGF- and EGFR-targeted drug therapies have been further explored. Although a large number of sequencing studies have previously revealed associations between specific gut microbial species or functions and colon cancer (Dejea et al., 2014; Kohoutova et al., 2014), the predictive power of particular cohorts and different colon sites has not been confirmed.

To further study the relationship between the intestinal microbiota and the colon cancer site, we collected tumor tissue and fecal samples from patient at Xiamen, which is representative of southern cities in China, and Harbin, which is representative of northern cities in China. We then compared the microbial community profiles in LCC and RCC and performed the first combined analysis of these profiles with different geographical regions to clarify the relationship between the intestinal microbiota and the etiology of colon cancer. Our new insights can better explain the morbidity of colon cancer, and the combination with targeted drug therapy might provide targets for the prevention of colon cancer or intervention strategies for this disease.

## Materials and Methods

### Sample Collection

Samples from forty patients diagnosed with colon cancer (19 at Zhongshan Hospital affiliated with Xiamen University, and 21 at the First Affiliated Hospital of Harbin Medical University) were collected in this study, and these samples included samples from the patient’s tumor tissue and feces.

Colon cancer tissues were collected when the patient underwent colon surgery and were stored at -80°C until DNA extraction. The stool sample was acquired by the patients themselves when they were notified by the doctor. Thus, the fecal samples were self-collected and sent to the laboratory within 1 h after excretion for storage until DNA extraction. All human materials were obtained with informed consent and approved by the ethics committees of Zhongshan Hospital affiliated with Xiamen University and the Hospital of Harbin Medical University.

### DNA Extraction and PCR Amplification

Microbial DNA was extracted using the HiPure Stool DNA Kit (Magen, Guangzhou, China) according to the manufacturer’s recommended protocols. The 16S rDNA V3-V4 region of the ribosomal RNA gene was amplified by PCR (95°C for 2 min, 27 cycles at 98°C for 10 s, 62°C for 30 s, and 68°C for 30 s, and a final extension at 68°C for 10 min) using the primers 341F (CCTACGGGNGGCWGCAG) and 806R (GGACTACHVGGGTATCTAAT), and the barcode was an eight-base sequence unique to each sample. The PCRs were performed in triplicate in a 50-μl mixture containing 5 μl of 10× KOD buffer, 5 μl of 2.5 mM dNTPs, 1.5 μl of each primer (5 μM), 1 μl of KOD polymerase, and 100 ng of template DNA.

### Illumina HiSeq 2500 Sequencing

Amplicons were extracted from 2% agarose gels, purified using the AxyPrep DNA Gel Extraction Kit (Axygen Biosciences, Union City, CA, USA) according to the manufacturer’s instructions and quantified using the ABI StepOnePlus Real-Time PCR System (Life Technologies, Foster City, Ca, USA). The purified amplicons were pooled in equimolar amounts and paired-end sequenced (2 × 250) with the Illumina platform according to standard protocols. The raw reads were deposited into the NCBI Sequence Read Archive (SRA) database (Accession Number: SRA: SRP258771 and Bioproject PRJNA628032).

### Quality Control and Read Assembly

Raw data containing adaptors or low-quality reads would affect the subsequent assembly and analysis. Thus, to obtain high-quality clean reads, the raw reads were further filtered according to the following rules using FASTP: reads containing more than 10% of unknown nucleotides-(N) and reads with less than 60% of bases with a quality value (Q-value) >20 were removed. Paired-end clean reads were merged as raw tags using FLSAH (Magoc and Salzberg, 2011) with a minimum overlap of 10 bp and a mismatch error rate of 2%. The noisy sequences of raw tags were filtered using the QIIME (Caporaso et al., 2010) pipeline based on specific filtering conditions (Bokulich et al., 2013) to obtain high-quality clean tags. The clean tags were searched against the reference database to perform reference-based chimera checking using the UCHIME algorithm. All chimeric tags were removed, and the final effective tags were used for further analysis.

### Sequence Analysis

The valid tags were clustered into operational taxonomic units (OTUs) with at least 97% similarity using the UPARSE pipeline (Edgar, 2013). The tag sequence with the highest abundance was selected as the representative sequence within each cluster. For the analyses between groups, Venn diagram-based analyses were performed in the R project to identify unique and common OTUs. The representative sequences were classified into organisms based on a naïve Bayesian model with the RDP classifier (Wang et al., 2007) using the SILVA database (Pruesse et al., 2007) with confidence threshold values ranging from 0.8 to 1. The abundance statistics of each taxon were visualized using Krona (Ondov et al., 2011). Biomarker features in each group were screened using MetaStats (White et al., 2009). Chao1, ACE, and all other alpha diversity indices were calculated with QIIME. The OTU rarefaction curve and rank abundance curves were plotted with QIIME. Comparisons of the alpha indexes between groups were performed with Welch’s t-test and Wilcoxon rank test using the R project. The comparisons of the alpha indexes among the groups were performed by Tukey’s HSD test and the Kruskal-Wallis H test using the R project. Sequence alignment was performed using Muscle (Manuel, 2013), and the phylogenetic tree was constructed using FastTree (Price et al., 2010). Weighted UniFrac distance matrixes were then generated using the GUniFrac package in the R project. The R project was also used to analyze the data based on multivariate statistical techniques, including principal component analysis (PCA), principal coordinates analysis (PCoA) and nonmetric multidimensional scaling (NMDS) of weighted UniFrac distances, and for plotting the results. Welch’s t-test, Wilcoxon rank test, and ANOSIM analysis were performed using the R project, and the KEGG pathway analysis of the OTUs was inferred using Tax4Fun (Asshauer et al., 2015).

## Results

### Dominant Species in the Microbiota of Colon Cancer Samples Belonging to Different Groups

We evaluated the communities of adherent bacteria in the mucosal tissue and fetal samples from 40 patients (21 from Harbin and 19 from Xiamen). We also analyzed several factors associated with colon cancer and found that only fecal occult blood tests showed statistically significant findings. The detailed characteristics of the subjects are described in **Table 1**. We first divided the collected tissue and stool samples into four groups according to the colon cancer location and the region at which the samples were collected: all cases of colon cancer can be divided into total LCC and total RCC (hereinafter referred to as total left and total right, respectively); the cases from Xiamen can also be divided into LCC and RCC; the cases from Harbin can be divided into LCC and RCC; and the different regions were divided into colon cancer cases from Xiamen and colon cancer cases from Harbin. The 16S rDNA gene sequencing method was used to analyze whether the differences among these subgroups affected the distribution of the gut microbiota.

**Table 1:** Correlation between different colon sites and clinicopathological characteristics of patients with colon cancer.

Parameter	Case	Colon sites		P value*
		Left	Right
Gender
Female	19	8	11	0.3422
Male	21	12	9	
Age (years)
≤65	19	10	9	0.7515
>65	21	10	11
Chemotherapy
No	20	9	11	0.5271
Yes	20	11	9	
TNM stage
I–II	25	13	12	0.7440
III–IV	15	7	8
Lymph node metastasis
No	25	12	13	0.7440
Yes	15	8	7
Fecal occult blood test
Positive	25	17	8	**0.0053**
Negative	14	3	11	

*P values are based on chi-square statistics for categorical variables. Bolded Text: P value less than 0.05 is statistically different.

**Figure 1:**
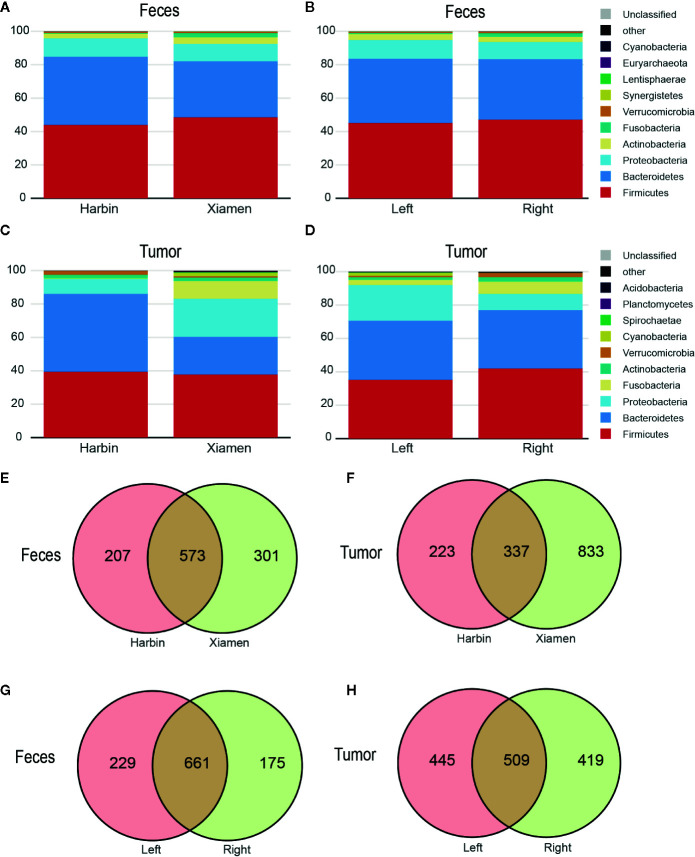
The microbial profile shows differences among the different groups. OTUs with at least 97% similarity within either patient group were classified at the phylum taxonomic level. Each bar represents the fecal or tumor microbial composition between the different groups. **(A)** Fecal and **(C)** tumor microbe composition in Harbin and Xiamen. **(B)** Fecal and **(D)** tumor microbe composition in the left- and right-sided colon samples. The scaled Venn diagrams prepared based on the fecal **(E)** and tumor samples **(F)** show the number of OTUs shared among the Harbin patients (pink), the Xiamen patients (yellow), and both groups (brown). The Venn diagrams prepared based on the fecal **(G)** and tumor samples **(H)** show the number of OTUs in LCC (pink), RCC (yellow), or both (brown).

The top four phyla in the fecal samples from Harbin and Xiamen were *Firmicutes*, *Bacteroidetes*, *Proteobacteria*, and *Actinobacteria*, and the fifth most abundant phyla in the samples from Harbin and Xiamen were *Verrucomicrobia* (0.82%) and *Fusobacteria* (2.52%), respectively (**Figure 1A**, **Table 2**). The five most abundant phyla in the left- and right-sided colon samples were the same and consisted of *Firmicutes*, *Bacteroidetes, Proteobacteria*, *Actinobacteria*, and *Fusobacteria* (**Figure 1B**), but the abundances of each bacterial phylum showed differences, as shown in **Table 3**. The top five phyla in the tumor tissue samples from Harbin and Xiamen included *Bacteroidetes*, *Firmicutes*, *Proteobacteria*, and *Actinobacteria* and either *Verrucomicrobia* (2.34%, in the Harbin samples) or *Fusobacteria* (10.55%, in the Xiamen samples) (**Figure 1C**, **Table 4**). The specificity of the latter two species in the tumor samples is consistent with the results from the fecal samples. The comparison of the left- and right-sided colon samples revealed that *Bacteroidetes*, *Firmicutes*, *Proteobacteria*, and *Fusobacteria* were among the top five phyla, and the last phyla identified in the left- and right-sided colon samples was *Cyanobacteria* (1.71%) and *Actinobacteria* (2.64%), respectively (**Figure 1D**, **Table 5**). The data obtained from the tumor samples from left and right sides of the colon showed more diversity, which was inconsistent with the results obtained from the fecal samples (**Tables 3**, **5**). The ratio abundance values were similar to the abundance values obtained in previous studies of the gut microbiota. We then identified the microflora by sequencing and clustered the sequences into OTUs with at least 97% similarity.

**Table 2:** Relative abundances of microbe species in the fecal samples from Harbin and Xiamen. (Species number of tags/total number of tags).

Phylum	Harbin	Xiamen
Firmicutes	4.41E+01	4.85E+01
Bacteroidetes	4.07E+01	3.36E+01
Proteobacteria	1.10E+01	1.04E+01
Actinobacteria	2.73E+00	3.97E+00
Fusobacteria	4.45E-01	2.53E+00
Verrucomicrobia	8.26E-01	6.37E-01
Synergistetes	3.34E-02	2.76E-01
Lentisphaerae	1.06E-01	6.32E-02
Euryarchaeota	1.00E-02	5.72E-03
Cyanobacteria	9.25E-03	3.93E-03
Other	6.53E-03	1.01E-02
Unclassified	3.67E-04	8.58E-04

**Table 3:** Relative abundances of microbe species in the fecal samples from the left and right sides of the colon. (Species number of tags/total number of tags).

Phylum	Left	Right
Firmicutes	4.52E+01	4.71E+01
Bacteroidetes	3.85E+01	3.62E+01
Proteobacteria	1.12E+01	1.03E+01
Actinobacteria	3.52E+00	3.12E+00
Fusobacteria	6.58E-01	2.21E+00
Verrucomicrobia	6.14E-01	8.58E-01
Synergistetes	2.46E-01	5.16E-02
Lentisphaerae	7.25E-02	9.93E-02
Euryarchaeota	1.07E-02	5.32E-03
Cyanobacteria	7.68E-03	5.78E-03
Other	8.21E-03	8.22E-03
Unclassified	1.01E-03	1.95E-04

**Table 4:** Relative abundances of microbe species in the tumor samples from Harbin and Xiamen. (Species number of tags/total number of tags).

Phylum	Harbin	Xiamen
Firmicutes	3.94E+01	3.78E+01
Bacteroidetes	4.67E+01	2.25E+01
Proteobacteria	8.88E+00	2.28E+01
Fusobacteria	3.94E-01	1.06E+01
Actinobacteria	2.11E+00	2.13E+00
Verrucomicrobia	2.34E+00	8.23E-01
Cyanobacteria	1.23E-02	2.06E+00
Spirochaetae	5.86E-04	4.03E-01
Planctomycetes	3.43E-04	2.13E-01
Acidobacteria	5.49E-03	1.59E-01
Other	1.67E-01	4.90E-01
Unclassified	1.67E-04	5.00E-02

**Table 5:** Relative abundances of microbe species in the tumor samples from the left and right sides of the colon. (Species number of tags/total number of tags).

Phylum	Left	Right
Firmicutes	3.52E+01	4.20E+01
Bacteroidetes	3.55E+01	3.49E+01
Proteobacteria	2.13E+01	9.72E+00
Fusobacteria	3.09E+00	7.35E+00
Actinobacteria	1.58E+00	2.65E+00
Verrucomicrobia	8.62E-01	2.38E+00
Cyanobacteria	1.71E+00	2.60E-01
Spirochaetae	3.81E-01	2.13E-03
Planctomycetes	1.78E-01	2.46E-02
Acidobacteria	1.85E-02	1.38E-01
Other	1.82E-01	4.59E-01
Unclassified	2.85E-03	4.49E-02

To understand the OTU crossover between the different groups, we used a Venn diagram to indicate the differences among the groups according to information on the OTU abundance. The samples from Xiamen showed an increased OTU abundance compared with the samples from Harbin, and no differences were found between the fecal and tumor tissue samples (**Figures 1E, F**). Simultaneously, both sets of data showed that the OTU abundance in the left-sided colon samples was higher than that in the right-sided colon samples (**Figures 1G, H**).

### The Microbial Compositions in the Left- and Right-Sided Colon Samples Show Significant Differences

**Figure 2:**
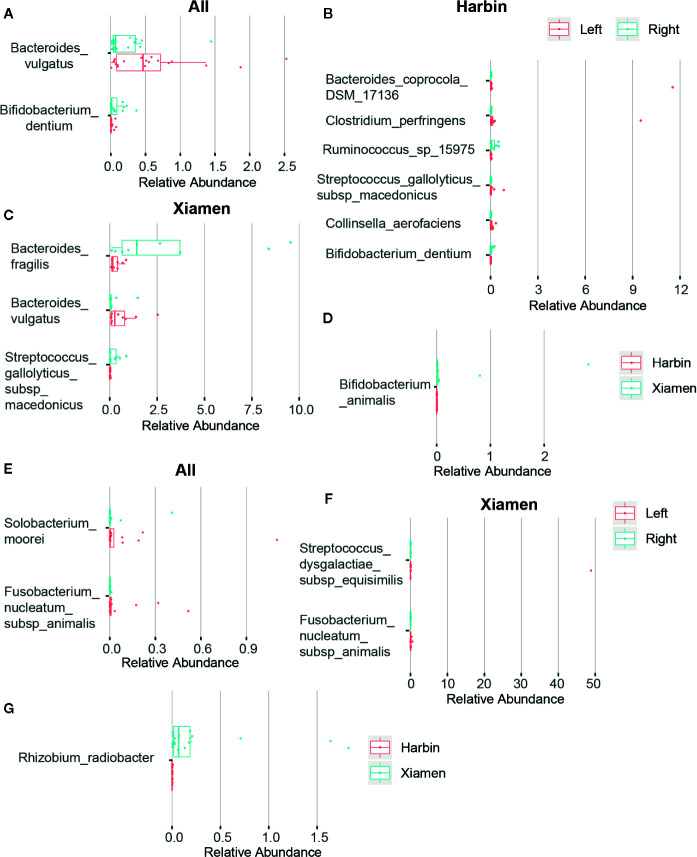
Differences in the microbial composition between LCC and RCC. The differences in the microbial composition and abundance between the different groups were tested using statistical methods (MetaStats). The graphic representation of the variations in the relative abundances of the species represented in different groups and the different OTUs are shown in rows. The differences in OTUs and taxa with p-values less than 0.05 are shown. Histograms obtained from comparing the relative OTU abundances between the left and right sides of the colon in the fecal **(A)** and tumor samples **(E)**, in the fecal samples obtained from Harbin **(B)** and Xiamen **(C)**, in the tumor samples obtained from Xiamen **(F)** and between Harbin and Xiamen in the fecal **(D)** and tumor samples **(G)**.

After obtaining a basic understanding of and classifying the species, we used statistical methods (MetaStats software, Wilcoxon rank sum test) to identify the differential species between pairs of the above-described groups. We analyzed the various species and discovered that the total area of *Bacteroides vulgatus* was significantly larger in the stool samples than in the right colon samples (*P <* 0.05), whereas *Bifidobacterium dentium* comprised a larger area in the right colon (*P <* 0.05) (**Figure 2A**, **Table 6**). The comparisons of the samples from a single region, such as Harbin, showed that *Clostridium perfringens*, *Bacteroides coprocola* DSM 17136, *Collinsella aerofaciens*, and *Streptococcus gallolyticus* subsp. *macedonicus* exhibited differences and were more highly enriched in the left side of the colon (*P <* 0.05) and that *B. dentium* and *Ruminococcus* sp. 15975 were highly present in the right side of the colon (*P <* 0.05) (**Figure 2B**, **Table 7**). In Xiamen, *B. vulgatus* was more highly enriched in the left side of the colon (*P <* 0.05), whereas *Bacteroides fragilis* (*P <* 0.05) and *S. gallolyticus* subsp. *macedonicus* (*P <* 0.01) were found at a higher abundance in the right compared with the left side of the colon (**Figure 2C**, **Table 8**). We subsequently compared all the microbes in the two regions and found a total of 37 different microbes (*P <* 0.05) (**Supplementary Table 1**), and among these, *Bifidobacterium animalis* exhibited the greatest difference and was highly enriched in Xiamen (*P <* 0.001) (**Figure 2D**).

**Table 6:** Species in the stool samples showing differential abundance between the left and right sides of the colon.

Species	Left (Mean abundance)	Right (Mean abundance)	fold (M-right/M-left)	p-value	q-value
Bacteroides_vulgatus	5.65E-01	2.30E-01	4.07E-01	1.55E-02	7.89E-01
Bifidobacterium_dentium	1.37E-02	6.58E-02	4.81E+00	3.83E-02	9.62E-01
human_gut_metagenome	4.49E-01	1.25E-01	2.78E-01	8.10E-02	9.62E-01
Bacteroides_coprocola_DSM_17136	1.17E+00	1.45E-01	1.24E-01	8.58E-02	9.62E-01
Prevotella_intermedia	5.23E-01	4.96E-02	9.48E-02	1.20E-01	9.62E-01
Lactobacillus_salivarius	1.80E-02	9.91E-02	5.51E+00	1.23E-01	9.62E-01
Ruminococcus_sp_15975	1.23E-02	8.51E-02	6.91E+00	1.51E-01	9.62E-01
Porphyromonas_asaccharolytica	7.02E-02	2.48E-03	3.54E-02	2.00E-01	9.62E-01
Collinsella_aerofaciens	1.02E-01	8.04E-02	7.86E-01	2.08E-01	9.62E-01
Sutterella_wadsworthensis_3_1_45B	6.15E-02	2.59E-01	4.21E+00	2.97E-01	9.62E-01
Bacteroides_fragilis	5.71E-01	4.54E+00	7.95E+00	3.14E-01	9.62E-01
Odoribacter_sp_Marseille-P2698	1.44E-01	4.03E-03	2.79E-02	3.21E-01	9.62E-01
Ruminococcus_sp_N15MGS-57	4.18E-03	2.88E-02	6.90E+00	3.44E-01	9.62E-01
Shewanella_algae	6.35E-04	1.02E-02	1.61E+01	3.53E-01	9.62E-01
Clostridium_sp	5.65E-01	5.77E-01	1.02E+00	3.87E-01	9.62E-01
Acinetobacter_johnsonii	2.64E-03	2.72E-02	1.03E+01	4.35E-01	9.62E-01
scindens	1.88E-02	8.52E-02	4.53E+00	4.49E-01	9.62E-01
bacterium_endosymbiont_of_Onthophagus_Taurus	1.15E-02	4.43E-02	3.87E+00	4.63E-01	9.62E-01
Acinetobacter_radioresistens	1.10E-02	3.28E-01	2.98E+01	4.69E-01	9.62E-01
Bifidobacterium_animalis	1.87E-01	5.67E-03	3.04E-02	4.69E-01	9.62E-01
Bacteroides_eggerthii	5.42E-02	1.52E-02	2.81E-01	4.81E-01	9.62E-01
Bifidobacterium_bifidum	1.87E-02	3.37E-02	1.80E+00	5.11E-01	9.62E-01
Lactobacillus_mucosae	1.07E-01	8.89E-02	8.31E-01	5.42E-01	9.62E-01
Peptoclostridium_difficile	5.32E-02	6.55E-02	1.23E+00	5.42E-01	9.62E-01
Clostridium_perfringens	5.17E-01	4.29E-02	8.29E-02	5.52E-01	9.62E-01
Bacteroides_uniformis	2.18E+00	1.02E+00	4.69E-01	5.83E-01	9.62E-01
Streptococcus_anginosus_subsp_anginosus	6.30E-02	5.01E-02	7.96E-01	5.88E-01	9.62E-01
Clostridium_baratii	1.71E-02	3.73E-01	2.18E+01	5.93E-01	9.62E-01
infirmum	1.35E-03	7.64E-03	5.68E+00	6.23E-01	9.62E-01
Dialister_pneumosintes	6.32E-02	4.81E-02	7.60E-01	6.45E-01	9.62E-01
Bacteroides_thetaiotaomicron	1.39E+00	1.02E+00	7.36E-01	6.59E-01	9.62E-01
Streptococcus_gallolyticus_subsp_macedonicus	6.23E-02	1.01E-01	1.62E+00	6.95E-01	9.62E-01
Bacteroides_stercoris_ATCC_43183	1.04E-02	2.41E-03	2.31E-01	6.99E-01	9.62E-01
Alistipes_sp_AL-1	9.08E-01	8.31E-01	9.16E-01	7.15E-01	9.62E-01
Morganella_morganii_subsp_morganii	6.97E-02	9.00E-02	1.29E+00	7.18E-01	9.62E-01
Parabacteroides_distasonis	1.74E+00	2.40E+00	1.38E+00	7.18E-01	9.62E-01
Alistipes_sp_N15MGS-157	8.19E-03	6.26E-03	7.64E-01	7.33E-01	9.62E-01
gut_metagenome	1.15E-01	6.02E-02	5.26E-01	7.76E-01	9.62E-01
Bifidobacterium_longum_subsp_longum	3.82E-01	3.72E-01	9.74E-01	7.79E-01	9.62E-01
Bacteroides_plebeius_DSM_17135	1.02E+00	1.45E+00	1.42E+00	7.99E-01	9.62E-01
Parabacteroides_goldsteinii	5.28E-01	8.01E-01	1.52E+00	7.99E-01	9.62E-01
Clostridium_thiosulfatireducens	1.28E-03	2.72E-02	2.13E+01	8.07E-01	9.62E-01
Desulfovibrio_desulfuricans	1.20E-02	7.84E-03	6.53E-01	8.33E-01	9.62E-01
Acinetobacter_calcoaceticus	8.71E-03	1.72E-01	1.98E+01	8.52E-01	9.62E-01
Collinsella_sp_GD3	6.77E-03	3.10E-04	4.58E-02	8.53E-01	9.62E-01
Haemophilus_influenzae	9.82E-03	2.08E-03	2.12E-01	8.67E-01	9.62E-01
Prevotella_buccae_D17	1.50E-01	4.16E-01	2.76E+00	8.87E-01	9.63E-01
Megasphaera_micronuciformis	3.58E-02	5.16E-03	1.44E-01	9.11E-01	9.68E-01
Ruminococcus_sp_UNKMGS-30	1.03E-01	1.03E-01	9.95E-01	9.46E-01	9.85E-01
Parabacteroides_faecis	1.46E-02	2.06E-02	1.41E+00	9.89E-01	1.00E+00
bacterium_NLAE-zl-G313	4.39E-02	8.50E-04	1.94E-02	1.00E+00	1.00E+00

**Table 7:** Species in the stool samples from Harbin showing differential abundance between the left and right sides of the colon.

Species	Left (Mean abundance)	Right (Mean abundance)	fold (M-right/M-left)	p-value	q-value
Clostridium_perfringens	1.02E+00	2.16E-02	2.11E-02	1.27E-02	2.73E-01
Bacteroides_coprocola_DSM_17136	1.18E+00	2.04E-02	1.73E-02	2.21E-02	2.73E-01
Collinsella_aerofaciens	7.42E-02	5.31E-03	7.16E-02	2.62E-02	2.73E-01
Bifidobacterium_dentium	1.30E-03	4.84E-02	3.72E+01	3.05E-02	2.73E-01
Ruminococcus_sp_15975	1.63E-02	1.44E-01	8.84E+00	4.30E-02	2.73E-01
Streptococcus_gallolyticus_subsp_macedonicus	1.17E-01	2.76E-03	2.36E-02	4.31E-02	2.73E-01
Odoribacter_sp_Marseille-P2698	2.54E-02	0.00E+00	0.00E+00	6.43E-02	3.49E-01
gut_metagenome	3.63E-02	9.28E-02	2.56E+00	1.30E-01	5.76E-01
Lactobacillus_salivarius	1.10E-02	1.14E-01	1.03E+01	1.41E-01	5.76E-01
Prevotella_intermedia	9.67E-02	7.82E-04	8.09E-03	1.57E-01	5.76E-01
Bacteroides_vulgatus	5.12E-01	2.36E-01	4.61E-01	1.73E-01	5.76E-01
human_gut_metagenome	6.68E-01	1.68E-01	2.51E-01	1.97E-01	5.76E-01
scindens	2.63E-02	1.37E-01	5.22E+00	1.97E-01	5.76E-01
Bacteroides_fragilis	8.43E-01	5.74E+00	6.81E+00	2.82E-01	6.69E-01
Bacteroides_plebeius_DSM_17135	1.79E+00	2.15E+00	1.20E+00	2.82E-01	6.69E-01
Bacteroides_thetaiotaomicron	2.13E+00	8.80E-01	4.14E-01	2.82E-01	6.69E-01
Dialister_pneumosintes	9.48E-02	5.52E-02	5.82E-01	3.76E-01	8.17E-01
bacterium_endosymbiont_of_Onthophagus_Taurus	2.15E-02	8.05E-02	3.75E+00	4.12E-01	8.17E-01
Parabacteroides_distasonis	2.60E+00	3.68E+00	1.41E+00	4.26E-01	8.17E-01
Ruminococcus_sp_N15MGS-57	3.28E-03	4.94E-02	1.50E+01	4.30E-01	8.17E-01
Bifidobacterium_bifidum	5.80E-04	2.13E-02	3.67E+01	4.58E-01	8.28E-01
Bifidobacterium_longum_subsp_longum	2.69E-01	4.75E-01	1.76E+00	5.57E-01	9.63E-01
Lactobacillus_mucosae	3.46E-02	6.50E-02	1.88E+00	6.18E-01	9.65E-01
Porphyromonas_asaccharolytica	1.40E-01	4.39E-03	3.13E-02	6.18E-01	9.65E-01
Acinetobacter_calcoaceticus	1.73E-02	3.13E-01	1.81E+01	6.47E-01	9.65E-01
Parabacteroides_faecis	2.46E-03	2.68E-02	1.09E+01	6.67E-01	9.65E-01
Parabacteroides_goldsteinii	9.54E-01	1.36E+00	1.42E+00	7.05E-01	9.65E-01
Peptoclostridium_difficile	9.87E-02	9.88E-02	1.00E+00	7.56E-01	9.65E-01
Streptococcus_anginosus_subsp_anginosus	6.93E-02	6.61E-02	9.53E-01	7.56E-01	9.65E-01
bacterium_NLAE-zl-G313	8.78E-02	1.55E-03	1.76E-02	8.03E-01	9.65E-01
Bacteroides_uniformis	2.76E+00	9.75E-01	3.53E-01	8.09E-01	9.65E-01
Ruminococcus_sp_UNKMGS-30	2.72E-02	7.37E-02	2.70E+00	8.33E-01	9.65E-01
Alistipes_sp_AL-1	1.78E-01	5.23E-01	2.93E+00	8.60E-01	9.65E-01
Sutterella_wadsworthensis_3_1_45B	1.20E-01	3.62E-01	3.02E+00	8.63E-01	9.65E-01
Acinetobacter_johnsonii	5.27E-03	4.90E-02	9.30E+00	9.15E-01	9.93E-01
Clostridium_sp	7.89E-01	9.00E-01	1.14E+00	9.73E-01	1.00E+00
Acinetobacter_radioresistens	2.20E-02	5.96E-01	2.70E+01	1.00E+00	1.00E+00
Morganella_morganii_subsp_morganii	1.39E-01	1.64E-01	1.18E+00	1.00E+00	1.00E+00

**Table 8:** Species in the stool samples from Xiamen showing differential abundance between the left and right sides of the colon.

Species	Left (Mean abundance)	Right (Mean abundance)	fold (M-right/M-left)	p-value	q-value
Streptococcus_gallolyticus_subsp_macedonicus	7.55E-03	2.22E-01	2.93E+01	7.94E-03	2.03E-01
Bacteroides_fragilis	2.99E-01	3.07E+00	1.03E+01	1.01E-02	2.03E-01
Bacteroides_vulgatus	6.18E-01	2.23E-01	3.61E-01	2.79E-02	3.72E-01
Clostridium_baratii	3.14E-02	8.27E-01	2.63E+01	1.33E-01	9.31E-01
Clostridium_sp	3.41E-01	1.83E-01	5.37E-01	1.82E-01	9.31E-01
Desulfovibrio_desulfuricans	2.34E-02	7.00E-04	2.99E-02	2.27E-01	9.31E-01
Bacteroides_eggerthii	1.02E-01	2.82E-02	2.77E-01	2.36E-01	9.31E-01
human_gut_metagenome	2.30E-01	7.21E-02	3.14E-01	2.43E-01	9.31E-01
gut_metagenome	1.93E-01	2.02E-02	1.05E-01	2.43E-01	9.31E-01
Sutterella_wadsworthensis_3_1_45B	3.09E-03	1.32E-01	4.27E+01	2.51E-01	9.31E-01
Lactobacillus_salivarius	2.50E-02	8.14E-02	3.26E+00	3.07E-01	9.31E-01
Bifidobacterium_dentium	2.60E-02	8.70E-02	3.34E+00	3.15E-01	9.31E-01
Streptococcus_anginosus_subsp_anginosus	5.66E-02	3.06E-02	5.40E-01	3.15E-01	9.31E-01
Clostridium_thiosulfatireducens	2.55E-03	6.05E-02	2.37E+01	3.26E-01	9.31E-01
Prevotella_intermedia	9.50E-01	1.09E-01	1.15E-01	3.56E-01	9.50E-01
Shewanella_algae	1.27E-03	2.27E-02	1.79E+01	4.70E-01	9.52E-01
Bifidobacterium_animalis	3.73E-01	1.24E-02	3.34E-02	4.97E-01	9.52E-01
Collinsella_aerofaciens	1.30E-01	1.72E-01	1.32E+00	4.97E-01	9.52E-01
Haemophilus_influenzae	1.96E-02	4.62E-03	2.35E-01	5.02E-01	9.52E-01
Bacteroides_uniformis	1.59E+00	1.08E+00	6.77E-01	6.04E-01	9.52E-01
Parabacteroides_distasonis	8.75E-01	8.23E-01	9.40E-01	6.04E-01	9.52E-01
Lactobacillus_mucosae	1.80E-01	1.18E-01	6.58E-01	6.24E-01	9.52E-01
Bacteroides_thetaiotaomicron	6.55E-01	1.20E+00	1.83E+00	6.61E-01	9.52E-01
Ruminococcus_sp_UNKMGS-30	1.79E-01	1.38E-01	7.72E-01	6.83E-01	9.52E-01
Clostridium_perfringens	9.36E-03	6.89E-02	7.36E+00	7.44E-01	9.52E-01
Alistipes_sp_N15MGS-157	1.38E-02	5.87E-03	4.25E-01	7.62E-01	9.52E-01
Megasphaera_micronuciformis	6.94E-02	6.49E-03	9.35E-02	7.75E-01	9.52E-01
Alistipes_sp_AL-1	1.64E+00	1.21E+00	7.38E-01	7.80E-01	9.52E-01
Bacteroides_coprocola_DSM_17136	1.15E+00	2.98E-01	2.58E-01	7.80E-01	9.52E-01
Bacteroides_plebeius_DSM_17135	2.60E-01	5.99E-01	2.30E+00	7.80E-01	9.52E-01
Parabacteroides_goldsteinii	1.03E-01	1.22E-01	1.18E+00	7.80E-01	9.52E-01
infirmum	1.94E-03	1.67E-02	8.62E+00	8.32E-01	9.52E-01
Prevotella_buccae_D17	2.99E-01	9.20E-01	3.08E+00	8.42E-01	9.52E-01
Collinsella_sp_GD3	1.35E-02	6.89E-04	5.09E-02	8.60E-01	9.52E-01
Bacteroides_stercoris_ATCC_43183	1.75E-02	3.93E-03	2.25E-01	9.01E-01	9.52E-01
Bifidobacterium_bifidum	3.66E-02	4.87E-02	1.33E+00	9.02E-01	9.52E-01
Parabacteroides_faecis	2.67E-02	1.29E-02	4.84E-01	9.02E-01	9.52E-01
Bifidobacterium_longum_subsp_longum	4.95E-01	2.47E-01	4.98E-01	9.05E-01	9.52E-01
Odoribacter_sp_Marseille-P2698	2.63E-01	8.94E-03	3.39E-02	9.67E-01	9.67E-01
Dialister_pneumosintes	3.16E-02	3.94E-02	1.25E+00	9.67E-01	9.67E-01

We also performed a statistical analysis of the differences in the tumor tissues. The total area of *Solobacterium moorei* and *Fusobacterium nucleatum* subsp. *animalis* was significantly larger in the left compared with the right side of the colon (*P <* 0.05) (**Figure 2E**, **Table 9**). In addition, *Streptococcus dysgalactiae* subsp. *equisimilis* and *F. nucleatum* subsp. *animalis* in Xiamen were more highly enriched in the left compared with right side of the colon (*P <* 0.01) (**Figure 2F**, **Table 10**). Similarly, the differences in the microbial population distribution due to geographical differences were substantial, and a total of 33 different microbes were found in both regions (*P <* 0.05) (**Supplementary Table 2**). Moreover, among the microbes found in both regions, *Rhizobium radiobacter* exhibited the most significant difference and was more highly enriched in Xiamen (*P <* 0.001) (**Figure 2G**). However, the tumor tissue samples from Harbin exhibited no difference between the left and right sides of the colon (data not shown).

**Table 9:** Species in the tumor samples showing differential abundance between the left and right sides of the colon.

Species	Left (Mean abundance)	Right (Mean abundance)	fold (M-right/M-left)	p-value	q-value
Fusobacterium_nucleatum_subsp_animalis	5.37E-02	5.20E-04	9.69E-03	2.37E-03	1.99E-01
Solobacterium_moorei	8.49E-02	2.46E-02	2.89E-01	2.03E-02	8.18E-01
Streptococcus_gallolyticus_subsp_macedonicus	2.35E-01	1.38E-02	5.86E-02	7.68E-02	8.18E-01
Lactobacillus_aviarius	0.00E+00	5.65E-03	Inf	8.06E-02	8.18E-01
Streptococcus_dysgalactiae_subsp_equisimilis	2.45E+00	1.59E-02	6.49E-03	8.59E-02	8.18E-01
Klebsiella_oxytoca	3.58E-02	2.03E-03	5.66E-02	1.30E-01	8.18E-01
Lachnospiraceae_bacterium_615	6.87E-02	7.11E-03	1.03E-01	1.36E-01	8.18E-01
Dialister_pneumosintes	1.53E-01	4.34E-02	2.84E-01	1.51E-01	8.18E-01
Pseudomonas_pertucinogena	9.55E-03	0.00E+00	0.00E+00	1.63E-01	8.18E-01
Collinsella_sp_GD3	5.44E-03	0.00E+00	0.00E+00	1.63E-01	8.18E-01
Lactobacillus_fermentum	5.15E-04	1.55E-02	3.01E+01	1.65E-01	8.18E-01
Campylobacter_gracilis	5.03E-02	4.22E-02	8.40E-01	1.77E-01	8.18E-01
Lactobacillus_mucosae	1.97E-02	3.44E-02	1.74E+00	1.82E-01	8.18E-01
Klebsiella_variicola	5.90E-01	5.62E-01	9.52E-01	1.83E-01	8.18E-01
Porites_australiensis	1.86E-01	2.77E-03	1.49E-02	1.97E-01	8.18E-01
human_gut_metagenome	1.60E-02	1.00E-02	6.25E-01	2.18E-01	8.18E-01
gut_metagenome	4.55E-01	8.56E-02	1.88E-01	2.24E-01	8.18E-01
Bacteroides_vulgatus	1.75E-01	5.42E-02	3.10E-01	2.39E-01	8.18E-01
Streptococcus_mutans	7.00E-05	1.11E-02	1.58E+02	2.75E-01	8.18E-01
Prevotella_intermedia	1.03E+00	1.54E-01	1.50E-01	2.80E-01	8.18E-01
Bromus_tectorum	1.01E-01	1.60E-02	1.58E-01	2.93E-01	8.18E-01
Parabacteroides_distasonis	1.47E+00	2.23E+00	1.51E+00	3.04E-01	8.18E-01
Streptococcus_anginosus_subsp_anginosus	2.43E-01	6.95E-02	2.86E-01	3.07E-01	8.18E-01
Lactobacillus_murinus	8.26E-02	1.79E-02	2.16E-01	3.21E-01	8.18E-01
Stenotrophomonas_rhizophila	5.30E-04	5.64E-03	1.06E+01	3.24E-01	8.18E-01
Acinetobacter_radioresistens	1.80E-04	1.71E-02	9.49E+01	3.27E-01	8.18E-01
Bacteroides_coprocola_DSM_17136	8.54E-01	2.05E-01	2.40E-01	3.29E-01	8.18E-01
Odoribacter_sp_Marseille-P2698	2.39E-02	0.00E+00	0.00E+00	3.42E-01	8.18E-01
Treponema_denticola	1.85E-02	0.00E+00	0.00E+00	3.42E-01	8.18E-01
Treponema_succinifaciens_DSM_2489	1.75E-02	0.00E+00	0.00E+00	3.42E-01	8.18E-01
Lactobacillus_harbinensis	1.16E-02	0.00E+00	0.00E+00	3.42E-01	8.18E-01
Paraburkholderia_kururiensis_subsp_kururiensis	0.00E+00	1.10E-02	Inf	3.42E-01	8.18E-01
delta_proteobacterium_WX152	0.00E+00	9.50E-03	Inf	3.42E-01	8.18E-01
Lactobacillus_agilis	0.00E+00	5.94E-03	Inf	3.42E-01	8.18E-01
Ruminococcus_sp_UNKMGS-30	1.88E-02	3.75E-02	2.00E+00	3.43E-01	8.18E-01
Bacteroides_uniformis	1.73E+00	1.36E+00	7.85E-01	3.51E-01	8.18E-01
Parabacteroides_faecis	7.26E-03	2.00E-02	2.76E+00	3.96E-01	8.76E-01
Parabacteroides_goldsteinii	5.13E-01	1.16E+00	2.26E+00	4.04E-01	8.76E-01
Acinetobacter_sp_BFE41A	3.04E-01	1.86E-01	6.13E-01	4.07E-01	8.76E-01
Haemophilus_influenzae	2.67E-01	3.44E-02	1.29E-01	4.26E-01	8.94E-01
Bifidobacterium_adolescentis	9.25E-03	4.73E-03	5.11E-01	4.46E-01	9.14E-01
unidentified_eubacterium_clone_342	1.76E-02	1.53E-01	8.68E+00	4.73E-01	9.28E-01
Bacteroides_fragilis	2.03E+00	5.43E+00	2.67E+00	4.78E-01	9.28E-01
Porphyromonas_sp_oral_clone_P4GB_100_P2	1.38E-01	4.76E-03	3.44E-02	4.86E-01	9.28E-01
Haemophilus_haemolyticus	5.55E-02	5.55E-03	1.00E-01	4.99E-01	9.32E-01
Clostridium_baratii	2.26E-03	2.42E-02	1.08E+01	5.10E-01	9.32E-01
Acinetobacter_calcoaceticus	3.89E-02	1.20E-01	3.08E+00	5.27E-01	9.37E-01
Ruminococcus_sp_15975	2.72E-02	1.01E-01	3.71E+00	5.39E-01	9.37E-01
Bifidobacterium_dentium	1.52E-02	8.55E-02	5.64E+00	5.65E-01	9.37E-01
Bacillus_coagulans	9.74E-02	6.15E-04	6.31E-03	5.74E-01	9.37E-01
Clostridium_butyricum	5.48E-02	1.63E-02	2.97E-01	5.83E-01	9.37E-01
Sphingomonas_paucimobilis	6.98E-02	9.97E-02	1.43E+00	5.88E-01	9.37E-01
bacterium_YE57	1.85E-04	6.41E-03	3.46E+01	6.15E-01	9.37E-01
Acinetobacter_johnsonii	7.22E-02	9.68E-03	1.34E-01	6.16E-01	9.37E-01
Brachyspira_sp_NSH-25	1.73E-02	2.60E-03	1.50E-01	6.21E-01	9.37E-01
aldenense	7.61E-02	5.18E-02	6.81E-01	6.34E-01	9.37E-01
Solanum_torvum	1.71E-02	4.93E-03	2.89E-01	6.36E-01	9.37E-01
Sphingomonas_aurantiaca	7.77E-03	7.32E-03	9.41E-01	6.89E-01	9.89E-01
Bifidobacterium_longum_subsp_longum	1.33E-01	3.06E-01	2.30E+00	6.95E-01	9.89E-01
Parabacteroides_sp_D13	1.56E-02	2.96E-02	1.90E+00	7.51E-01	1.00E+00
Clostridium_perfringens	3.49E-01	2.14E-01	6.13E-01	7.54E-01	1.00E+00
Bifidobacterium_bifidum	1.14E-02	9.64E-02	8.43E+00	7.63E-01	1.00E+00
Bacteroides_eggerthii	3.02E-02	4.12E-03	1.36E-01	7.75E-01	1.00E+00
Porphyromonas_asaccharolytica	1.15E-01	6.40E-04	5.57E-03	7.76E-01	1.00E+00
Veillonella_parvula	1.94E-02	1.34E-02	6.91E-01	7.98E-01	1.00E+00
Clostridium_sp	5.09E-01	6.54E-01	1.28E+00	8.41E-01	1.00E+00
Bacteroides_plebeius_DSM_17135	1.46E+00	2.28E+00	1.56E+00	8.50E-01	1.00E+00
Bifidobacterium_breve	1.19E-02	2.35E-02	1.98E+00	8.59E-01	1.00E+00
Alistipes_sp_AL-1	2.20E-01	5.20E-01	2.37E+00	8.60E-01	1.00E+00
Parabacteroides_merdae	1.11E+00	1.82E+00	1.65E+00	8.70E-01	1.00E+00
bacterium_endosymbiont_of_Onthophagus_Taurus	1.20E-02	7.43E-03	6.16E-01	8.92E-01	1.00E+00
Lactobacillus_salivarius	1.36E-02	4.95E-02	3.65E+00	9.28E-01	1.00E+00
Pseudomonas_oryzihabitans	1.54E-02	8.55E-04	5.55E-02	9.31E-01	1.00E+00
Rhizobium_radiobacter	1.57E-01	1.11E-01	7.07E-01	9.34E-01	1.00E+00
scindens	1.45E-01	8.62E-02	5.93E-01	9.35E-01	1.00E+00
Bacteroides_thetaiotaomicron	2.11E+00	2.13E+00	1.01E+00	9.47E-01	1.00E+00
Flavobacterium_sp_YH1	1.37E-03	2.65E-02	1.94E+01	9.73E-01	1.00E+00
Bacillus_smithii	8.77E-02	1.58E-03	1.80E-02	9.79E-01	1.00E+00
leptum	2.18E-02	4.40E-04	2.02E-02	9.79E-01	1.00E+00
Eubacterium_ramulus	7.08E-02	1.52E-01	2.14E+00	9.89E-01	1.00E+00
Sphingobium_yanoikuyae	5.02E-02	2.70E-02	5.37E-01	1.00E+00	1.00E+00
bacterium_NLAE-zl-G313	5.10E-02	2.10E-04	4.12E-03	1.00E+00	1.00E+00
unidentified_rumen_bacterium_12-124	2.34E-02	3.85E-04	1.64E-02	1.00E+00	1.00E+00
mouse_gut_metagenome	1.27E-02	1.45E-04	1.14E-02	1.00E+00	1.00E+00

**Table 10:** Species in the tumor samples from Xiamen showing differential abundance between the left and right sides of the colon.

Species	Left (Mean abundance)	Right (Mean abundance)	fold (M-right/M-left)	p-value	q-value
Streptococcus_dysgalactiae_subsp_equisimilis	4.90E+00	3.68E-03	7.51E-04	2.86E-03	2.00E-01
Fusobacterium_nucleatum_subsp_animalis	1.06E-01	1.16E-03	1.09E-02	5.12E-03	2.00E-01
Lactobacillus_aviarius	0.00E+00	1.25E-02	Inf	6.24E-02	7.93E-01
Parabacteroides_goldsteinii	1.73E-02	3.70E-01	2.14E+01	9.23E-02	7.93E-01
Solobacterium_moorei	1.60E-01	5.33E-02	3.33E-01	9.34E-02	7.93E-01
Bromus_tectorum	2.03E-01	3.55E-02	1.75E-01	9.40E-02	7.93E-01
scindens	2.75E-01	2.19E-02	7.94E-02	1.01E-01	7.93E-01
Klebsiella_oxytoca	6.44E-02	1.79E-03	2.78E-02	1.01E-01	7.93E-01
Streptococcus_anginosus_subsp_anginosus	4.12E-01	1.16E-01	2.82E-01	1.29E-01	7.93E-01
Bacteroides_fragilis	2.72E+00	5.46E+00	2.01E+00	1.33E-01	7.93E-01
Bacteroides_vulgatus	1.64E-01	1.58E-02	9.66E-02	1.41E-01	7.93E-01
Lactobacillus_fermentum	0.00E+00	3.39E-02	Inf	1.46E-01	7.93E-01
Lachnospiraceae_bacterium_615	1.37E-01	1.50E-02	1.09E-01	1.71E-01	7.93E-01
Bacteroides_coprocola_DSM_17136	4.12E-01	4.47E-01	1.09E+00	1.87E-01	7.93E-01
Bacillus_coagulans	1.95E-01	0.00E+00	0.00E+00	1.93E-01	7.93E-01
Pseudomonas_pertucinogena	1.91E-02	0.00E+00	0.00E+00	1.93E-01	7.93E-01
Dialister_pneumosintes	1.79E-01	4.60E-02	2.57E-01	1.99E-01	7.93E-01
Lactobacillus_murinus	1.65E-01	3.93E-02	2.38E-01	2.27E-01	7.93E-01
Porites_australiensis	3.71E-01	6.16E-03	1.66E-02	2.44E-01	7.93E-01
Streptococcus_gallolyticus_subsp_macedonicus	4.07E-01	2.79E-02	6.86E-02	2.51E-01	7.93E-01
Lactobacillus_mucosae	2.56E-03	6.96E-02	2.72E+01	2.69E-01	7.93E-01
Acinetobacter_johnsonii	1.44E-01	2.08E-02	1.44E-01	2.70E-01	7.93E-01
Ruminococcus_sp_UNKMGS-30	1.72E-02	2.22E-04	1.29E-02	2.77E-01	7.93E-01
Bifidobacterium_dentium	2.71E-02	1.54E-01	5.69E+00	2.96E-01	7.93E-01
Bacteroides_eggerthii	5.34E-02	2.54E-03	4.77E-02	2.99E-01	7.93E-01
Porphyromonas_sp_oral_clone_P4GB_100_P2	2.77E-01	8.63E-03	3.12E-02	2.99E-01	7.93E-01
Alistipes_sp_AL-1	2.40E-01	3.62E-01	1.51E+00	3.03E-01	7.93E-01
Clostridium_perfringens	4.97E-03	4.72E-01	9.51E+01	3.03E-01	7.93E-01
Paraburkholderia_kururiensis_subsp_kururiensis	0.00E+00	2.44E-02	Inf	3.43E-01	7.93E-01
delta_proteobacterium_WX152	0.00E+00	2.11E-02	Inf	3.43E-01	7.93E-01
Lactobacillus_agilis	0.00E+00	1.32E-02	Inf	3.43E-01	7.93E-01
Campylobacter_gracilis	9.98E-02	9.38E-02	9.40E-01	3.79E-01	7.93E-01
Ruminococcus_sp_15975	5.50E-03	1.46E-02	2.65E+00	3.96E-01	7.93E-01
Porphyromonas_asaccharolytica	1.10E-01	0.00E+00	0.00E+00	3.99E-01	7.93E-01
Treponema_denticola	3.71E-02	0.00E+00	0.00E+00	3.99E-01	7.93E-01
Treponema_succinifaciens_DSM_2489	3.50E-02	0.00E+00	0.00E+00	3.99E-01	7.93E-01
mouse_gut_metagenome	2.55E-02	0.00E+00	0.00E+00	3.99E-01	7.93E-01
Lactobacillus_harbinensis	2.32E-02	0.00E+00	0.00E+00	3.99E-01	7.93E-01
Collinsella_sp_GD3	1.07E-02	0.00E+00	0.00E+00	3.99E-01	7.93E-01
Acinetobacter_calcoaceticus	7.71E-02	2.23E-02	2.90E-01	4.07E-01	7.93E-01
Bacteroides_uniformis	1.51E-01	2.45E-01	1.61E+00	4.37E-01	8.11E-01
Haemophilus_influenzae	5.34E-01	7.45E-02	1.40E-01	4.45E-01	8.11E-01
Klebsiella_variicola	3.67E-01	1.44E-01	3.93E-01	4.47E-01	8.11E-01
Solanum_torvum	3.32E-02	8.58E-03	2.59E-01	4.84E-01	8.34E-01
Flavobacterium_sp_YH1	2.58E-03	5.89E-02	2.28E+01	4.90E-01	8.34E-01
Prevotella_intermedia	1.92E+00	3.42E-01	1.78E-01	4.97E-01	8.34E-01
Bifidobacterium_bifidum	2.27E-02	2.14E-01	9.41E+00	5.03E-01	8.34E-01
Stenotrophomonas_rhizophila	1.06E-03	1.23E-02	1.16E+01	5.20E-01	8.34E-01
unidentified_eubacterium_clone_342	7.95E-03	3.39E-01	4.26E+01	5.24E-01	8.34E-01
leptum	4.36E-02	1.78E-04	4.08E-03	5.63E-01	8.78E-01
unidentified_rumen_bacterium_12-124	4.68E-02	2.22E-04	4.74E-03	6.07E-01	9.28E-01
Parabacteroides_distasonis	1.25E-01	2.13E-01	1.70E+00	6.24E-01	9.36E-01
Veillonella_parvula	2.03E-02	5.56E-03	2.74E-01	6.74E-01	9.51E-01
Lactobacillus_salivarius	1.48E-02	6.99E-02	4.72E+00	6.83E-01	9.51E-01
Bifidobacterium_breve	2.36E-02	5.01E-02	2.12E+00	6.83E-01	9.51E-01
Eubacterium_ramulus	3.11E-02	2.59E-01	8.35E+00	7.06E-01	9.51E-01
gut_metagenome	9.02E-01	9.77E-02	1.08E-01	7.09E-01	9.51E-01
Bacteroides_plebeius_DSM_17135	6.30E-01	3.25E+00	5.16E+00	7.20E-01	9.51E-01
Rhizobium_radiobacter	3.14E-01	2.45E-01	7.82E-01	7.20E-01	9.51E-01
Sphingobium_yanoikuyae	1.00E-01	5.99E-02	5.98E-01	7.72E-01	1.00E+00
Parabacteroides_merdae	9.14E-02	2.30E-01	2.51E+00	8.97E-01	1.00E+00
aldenense	9.40E-02	5.65E-02	6.02E-01	9.00E-01	1.00E+00
Sphingomonas_paucimobilis	1.39E-01	2.21E-01	1.59E+00	9.02E-01	1.00E+00
Clostridium_butyricum	1.07E-01	3.53E-02	3.30E-01	9.02E-01	1.00E+00
Bacteroides_thetaiotaomicron	4.76E-01	2.37E+00	4.98E+00	9.05E-01	1.00E+00
Acinetobacter_sp_BFE41A	6.06E-01	4.11E-01	6.78E-01	9.05E-01	1.00E+00
Streptococcus_mutans	1.40E-04	2.28E-02	1.63E+02	9.39E-01	1.00E+00
Parabacteroides_faecis	4.98E-03	1.58E-02	3.17E+00	9.39E-01	1.00E+00
bacterium_YE57	1.60E-04	1.42E-02	8.90E+01	9.39E-01	1.00E+00
bacterium_endosymbiont_of_Onthophagus_Taurus	4.90E-04	1.18E-02	2.40E+01	9.39E-01	1.00E+00
Clostridium_baratii	1.02E-03	5.05E-02	4.95E+01	9.54E-01	1.00E+00
Brachyspira_sp_NSH-25	2.69E-02	5.78E-03	2.15E-01	9.54E-01	1.00E+00
Sphingomonas_aurantiaca	1.55E-02	1.61E-02	1.03E+00	9.54E-01	1.00E+00
Pseudomonas_oryzihabitans	3.08E-02	1.90E-03	6.16E-02	9.61E-01	1.00E+00
Haemophilus_haemolyticus	1.10E-01	1.23E-02	1.12E-01	9.65E-01	1.00E+00
Clostridium_sp	3.20E-01	4.95E-01	1.54E+00	1.00E+00	1.00E+00
Bifidobacterium_longum_subsp_longum	6.96E-02	2.53E-01	3.64E+00	1.00E+00	1.00E+00
Bacillus_smithii	1.76E-01	3.51E-03	2.00E-02	1.00E+00	1.00E+00

### Comparisons of the Microbial Diversity Between the Left- and Right-Sided Colon Samples

**Figure 3:**
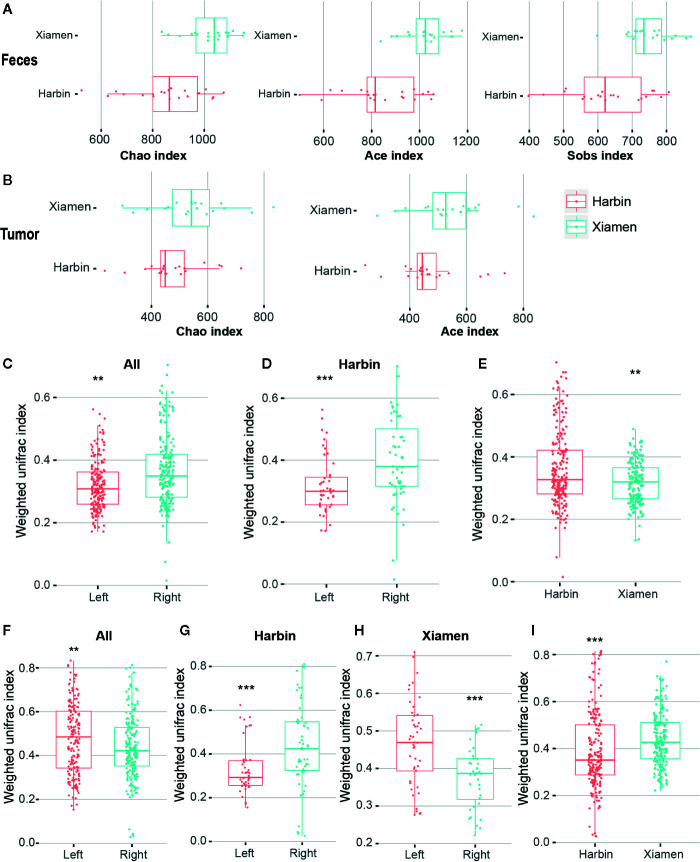
Diversity analysis of LCC and RCC. Qiime software was used to calculate the alpha diversity based on OTUs. **(A)** Alpha diversity indexes (Chao1, ACE and Sobs) obtained for the stool samples from Xiamen and Harbin with p-values less than 0.001. **(B)** Differences in the Chao1 and ACE indexes obtained for the tumor samples from Xiamen and Harbin exhibited p-values less than 0.05. A UPGMA cluster analysis was performed using the weighted UniFrac distance matrix. The average weighted UniFrac distance values (beta diversities) based on the fecal samples between the total left and right samples **(C)**, between the left and right samples from Harbin **(D)** and between Xiamen and Harbin **(E)** are shown. Statistically different differences were found between the total left and right samples **(F)**, between the left and right samples from Harbin **(G)**, between the left and right samples from Xiamen **(H)** and between Xiamen and Harbin **(I)**; the average weighted UniFrac distance values are shown. **P < 0.01, ***P < 0.001.

For comparisons of the microbial diversity within and between the samples belonging to different groups, we also performed a diversity analysis. Alpha diversity refers to the variety within a particular ecosystem and thus indicates the extent to which species isolate the system. We first calculated alpha diversity indexes (Chao1, ACE, Sobs, Shannon, and Simpson) and determined that three diversity richness estimators, namely, Chao1, ACE and Sobs, showed significant differences between the stool samples from Xiamen and those from Harbin (*P <* 0.001) (**Figure 3A**). However, the analysis of the tumor tissues revealed that only two diversity estimation indices, Chao1 and ACE, exhibited significant differences between the two regions (*P <* 0.05) (**Figure 3B**, **Supplementary Table 3**). In addition, no significant differences were obtained from the intrasample analysis of the remaining subgroups (data not shown). We used two classic beta diversity indexes, Jaccard distance index and Bray abundance index, and confirming that the grouped species have differences in bacterial structure and species abundance (**Supplementary Figures 1**, **2**). The impact of the region on the microbiota might be higher than the that of the distribution of the gut, and this effect might be related to differences in diet and the environment.

We performed a UniFrac analysis to initially determine the underlying factors driving changes in community diversity, and the analyses of the fecal samples identified differences in the bacterial community (using the Wilcoxon rank sum test and weighing all the data) between the total left and right samples (*P <* 0.01) (**Figure 3C**), between the left and right samples from Harbin (*P <* 0.001) (**Figure 3D**), and between the samples from Xiamen and those from Harbin (*P <* 0.01) (**Figure 3E**). In contrast, the analysis of the tumor tissue samples revealed significant differences in bacterial communities between the total left and right (*P <* 0.01) (**Figure 3F**), between the left and right samples from Harbin (**Figure 3G**), between the left and right samples from Xiamen (**Figure 3H**), and between the samples from Xiamen and those from Harbin (**Figure 3I**); in this analysis, the same Wilcox rank sum test was used, and all the data were weighted (*P <* 0.001, **Table 11**). A similarity matrix analysis (ANOSIM) also showed a significant difference in the bacterial composition between the different regions (*P <* 0.05, data not shown). These results indicated that when the presence or absence of a species and the species abundance are simultaneously considered, the species composition exhibits significant variation along the environmental gradient or between communities, which also indicates that the biological species show a greater difference in response to environmental heterogeneity.

**Table 11:** Beta diversities in different groups of fecal and tumor samples.

Division	Area	Group	P value
Feces	All	Left-VS-Right	1.92E-05
	Harbin	Left-VS-Right	3.23E-04
	Harbin-VS-Xiamen		2.17E-03
Tumor	All	Left-VS-Right	1.66E-02
	Harbin	Left-VS-Right	3.06E-04
	Xiamen	Left-VS-Right	1.60E-04
	Harbin-VS-Xiamen		1.58E-05

### Different Colon Cancer Locations Alter the Intestinal Microbiota Function

Several lines of evidence indicate that the functional composition of the microbiota is closely related to the species composition and environment. Due to the development of improved analytical techniques, the use of diversity sequencing data for microbiota function prediction has become essential in community research. We used the predictive software Tax4Fun to analyze the differences in intestinal microbiota functions between colon cancer at different sites.

**Figure 4:**
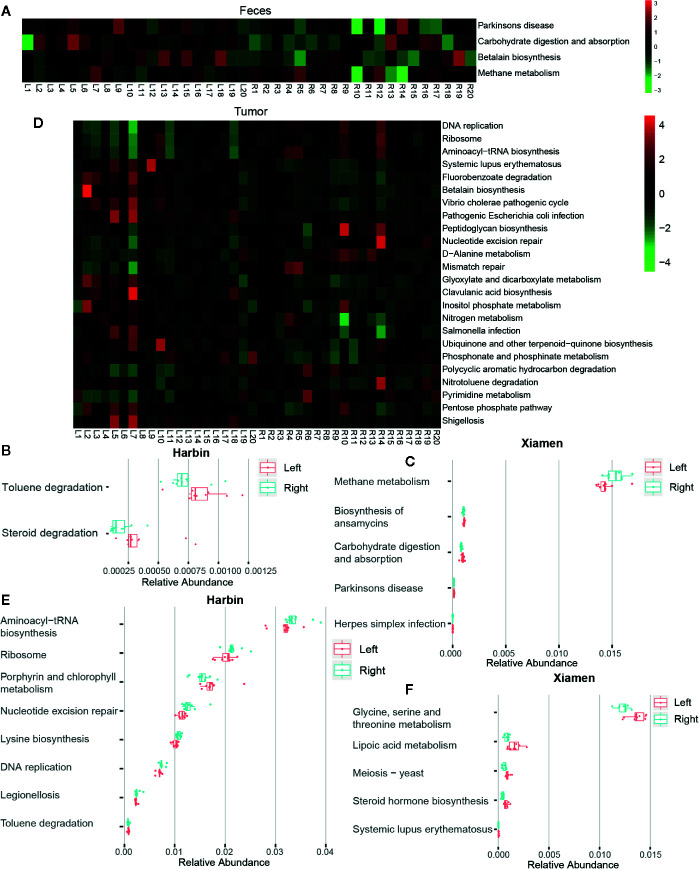
Tumor locations determine the intestinal microbiota function. The association of genetic modules with the colon cancer status was analyzed using Tax4fun, and KEGG pathways that showed differences among different groups were identified. Heat maps based on the results from the SILVA database with p-values less than 0.05 obtained from comparing the relative abundances between the total left and right fecal **(A)** and tumor samples **(D)** are shown. The subjects are shown in the columns, and the different pathways are shown in the rows. The green asterisks indicate OTUs with decreased proportions, whereas the red asterisks indicate OTUs with increased dimensions based on individual controls. Graphic representations of the differences in the relative abundances of microbial KEGG modules between the left and right fecal **(B)** and tumor samples **(E)** from Harbin, between the left and right fecal **(C)** and tumor samples **(F)** from Xiamen are shown.

A total of 284 KEGG pathways were generated by the analysis of the 16S rDNA gene sequencing data using Tax4Fun. The analysis of the fecal samples revealed that the intestinal microbiota with a higher abundance in the left compared with the right sides of the colon was significantly increased in pathways involved in carbohydrate digestion and absorption (*P <* 0.05), Parkinson’s disease (*P <* 0.05), and betalain biosynthesis (*P <* 0.05), with thez exception of methane metabolism (*P <* 0.05) (**Figure 4A**). The analysis of the fecal samples from Harbin showed that the microbiota species found at higher levels in the right compared with the left sides of the colon exhibited lower toluene degradation (*P <* 0.05) and steroid degradation (*P <* 0.01) (**Figure 4B**). In Xiamen, pathways associated with the biosynthesis of ansamycins, carbohydrate digestion and absorption, Parkinson’s disease and herpes simplex infection were more abundant in the fecal samples from the left compared with the right sides of the colon (*P <* 0.05), whereas methane metabolism was more highly enriched in right side of the colon (*P <* 0.05) (**Figure 4C**). The comparison between Xiamen and Harbin revealed eight functional changes, and the most notable among these were Epstein-Barr virus infection (*P <* 0.01), tight junction (*P <* 0.05), leukocyte transendothelial migration (*P <* 0.05), adherens junction (*P <* 0.05), and photosynthesis-antenna proteins (*P <* 0.05) (data not shown).

Similarly, we also analyzed the changes in tumor tissue sample. Unlike the results obtained from the fecal samples, 24 pathway-related differences in the microbial function predictions were found between LCC and RCC. Among these, the top five were DNA replication (*P <* 0.001), ribosome (*P <* 0.01), aminoacyl-tRNA biosynthesis (*P <* 0.01), systemic lupus erythematosus (*P <* 0.01), and fluorobenzoate degradation (*P <* 0.01) (**Figure 4D**). In Harbin, eight pathways showed differences between the left and right sides of the colon, and the most notable of these was nucleotide excision repair (*P <* 0.01) (**Figure 4E**). However, 33 pathways showed differences between the left- and right-sided colon samples from Xiamen, and the most prominent of these were steroid hormone biosynthesis (*P <* 0.001), meiosis-yeast (*P <* 0.001), glycine, serine and threonine metabolism (*P <* 0.01), lipoic acid metabolism (*P <* 0.01), and systemic lupus erythematosus (*P <* 0.01) (**Figure 4F**). We then compared Xiamen and Harbin and found differences in 157 pathways, and the top five of these were photosynthesis-antenna proteins, dilated cardiomyopathy, arrhythmogenic right ventricular cardiomyopathy (ARVC), cell adhesion molecules (CAMs), and the NF-kappa B signaling pathway (all the P values were less than 0.001, data not shown). The pathway-based differences in function found from the analysis of the tissue samples showed greater significance compared with those obtained from the analysis of the fecal samples.

## Discussion

The microbiota plays a vital role in the intestine, particularly in colon cancer (Gao et al., 2015; Wong and Yu, 2019). Most previous studies only analyzed the colorectal cancer-associated mucosal microbiota based on only fecal or tissue samples, and only few pairs of fecal and mucosal samples were studied (Zeller et al., 2014). Additionally, further exploration of the differences in the colon cancer sites and geographical regions is not possible. To perform a comprehensive analysis in this study, we collected fecal and tissue samples from patients in different regions to identify adherent bacteria and assessed whether colony differences in different parts of the intestine were associated with colonic carcinogenesis.

By combining the results from the fecal and tumor tissues, we found that the four major phyla, namely, Firmicutes, Bacteroidetes, Proteobacteria, and Actinobacteria, were found in both the total left and right samples, but in the tumor tissues, a higher abundance of Cyanobacteria was found in the left- compared with right-sided colon samples. The analyses of the different regions revealed that Fusobacteria was uniquely found in Xiamen and that Verrucomicrobia was abundant in Harbin. In addition, we also determined the degrees of bacterial enrichment in the fecal and tumor tissue samples belonging to the different groups. The flora richness in the samples from Xiamen and the left-sided colon samples was higher than that in the samples from Harbin and the right-sided colon samples. In summary, we found that Firmicutes, Bacteroidetes, Proteobacteria, and Actinobacteria are the main phyla in the colon, and this finding was also consistent with the results from previous studies of intestinal microecology (Eckburg et al., 2005; Louis et al., 2014). Because these species are resident bacteria in the gastrointestinal tract, the differences obtained in our research results revealed that changes in the composition and structure of the community of attached bacteria might contribute to the development of colon cancer. Of course, geographical differences and different eating habits cannot be excluded as potential reasons for the observed differences.

To identify the bacterial composition of the gastrointestinal tract and understand the role of bacteria in health and disease, which is currently a focus of ecological research, we further analyzed the species differences between the different groups. The flora in the feces and tissues show wide variations. For example, in feces, *B. vulgatus* exhibits higher expression in the left compared with the right sides of the colon, whereas *B. dentium* is more prominent in the right compared with the left sides of the colon. The consideration of geographical factors revealed that in Harbin, harmful bacteria, such as *C. perfringens*, *B. coprocola*, *C. aerofaciens*, and *S. gallolyticus*, which are associated with microscopic inflammation, are more highly enriched in the left compared with right sides of the colon cancer, whereas *B. dentium* was more abundant in the right compared with left sides of the colon. In Xiamen, *B. vulgatus* showed in the left-sided colon samples. In addition, *B. fragilis* and *S. gallolyticus* were more highly enriched in right compared with left sides of the colon. The analysis of tissue samples showed that *S. moorei* and *F. nucleatum* were more abundant in the left compared with the right sides of the colon. Moreover, in Xiamen, *F. nucleatum* and *S. dysgalactiae* were more prominent in the left compared with the right sides of the colon. Furthermore, the samples from Xiamen and Harbin exhibit different expression levels of *B. animalis*, which plays a protective role in the colon, and *R. radiobacter*.

Based on previous reports, *F. nucleatum* and excess *B. coprocola* accelerate the onset of colon tumors and promote the transition of the environment to a proinflammatory microenvironment that favors colorectal tumorigenesis, these also indicate that the bacteria themselves and their metabolites will affect the sensitivity of the drug (Kostic et al., 2012; Kostic et al., 2013; Tahara et al., 2014). *B. fragilis*, *S. gallolyticus*, and *Solobacterium* are closely related to colorectal cancer and are considered harmful bacteria (Kwong et al., 2018). In addition, *C. perfringens* and *C. aerofaciens* can degenerate proteins to produce toxins, which not only cause food poisoning but also produce carcinogens (Eichner et al., 2017). In contrast, *Bifidobacterium* can promote intestinal peristalsis, digestion and absorption and enhance the vitality of immune cells (Walker et al., 2011). Benedix et al. reported differences in the incidence of ethnic groups, and a higher proportion of LCC is found in the Asian populations (Benedix et al., 2010b). Based on our investigation, these findings might be explained by the type and number of pathogenic bacteria in the left-sided colon samples. Our results indicate a massive difference in flora between the left- and right-sided colon samples. Specifically, the microbiota in the left-sided colon samples is more likely to aggravate colon cancer, whereas the flora in the right-sided colon samples exhibits less invasiveness, decreased harmfulness, and protects a few features, with the exception of *B. fragilis* and *S. gallolyticus*, which were found to be expressed in the samples from Xiamen. In addition, the samples from Xiamen tended to express more beneficial bacteria than those from Harbin, which might be related to the differences in diet and living environments between South and North China.

Based on the responses of the microbial communities to current individualized drug therapies (Geller et al., 2017; Yu et al., 2017; Koh et al., 2020), including the currently prevalent chemotherapies using targeted drugs for VEGF and EGFR, we hypothesized that the flora found at different tumor sites will affect the clinical treatment decisions. *Clostridium difficile* induces VEGF-A and vascular permeability to promote disease pathogenesis (Huang et al., 2019), and *F. nucleatum* infection increases the level of VEGF release after 12 h (Mendes et al., 2016). Our results indicate that *Clostridium* and *Fusobacterium nucleatum* are highly enriched in the left side of the colon and might be related to the expression of VEGF, which indicates that the integration of treatments using the VEGF-targeted drug bevacizumab would be beneficial. In addition, Andrew W et al. found that *Bacteroides* antibiotics combined with VEGF tyrosine kinase inhibitors significantly improve the efficacy of metastatic renal cell carcinoma (Hahn et al., 2018). We found that *B. vulgatus* and *B. fragilis* are expressed in the left and right sides of the colon, respectively. Interestingly, these results are consistent with the responses to the current treatment for colon cancer involving bevacizumab and further indicate that the addition of antibiotics might improve the effect of bevacizumab on patients with a high abundance of *Bacteroides*. However, few studies have investigated EGFR, and these have confirmed that EGFR might be an essential host target for further research on the prevention of neuroinflammation caused by *Streptococcus suis* serotype 2 (SS2) (Yang et al., 2016). Combined with our study, we found that *Streptococcus* is expressed in both the left and right sides of the colons and is more highly enriched in the left side of the colon, including in the feces and tumor tissues. At present, the international discussion on the EGFR-targeting drug cetuximab reveals that most scholars believe that EGFR monoclonal antibody is more therapeutic for the overall survival of patients with LCC and could be used as a first-line optimized treatment for LCC. However, treatment decisions should consider the patient’s age, underlying disease, primary lesions, quality of life, and other comprehensive assessments to manage the patients throughout the process.

Due to the development of analytical techniques, the use of diversity sequencing data for community function prediction has become vital in microflora research. We predicted the KEGG functional modules using Tax4Fun and the SILVA database, and the study confirmed changes in 284 pathways. Overall, the main functional pathways involved in LCC are toluene, steroid and fluorobenzoate degradation, carbohydrate digestion and absorption, lipoic acid, glycine, serine and threonine metabolism, betalain, ansamycins, and steroid hormone biosynthesis. These biological processes are primarily associated with genetic mutations and epigenetic changes that mediate DNA damage and methylation, histone modifications, and an immune disorder (Ellmerich et al., 2000; Goodwin et al., 2011; Kostic et al., 2013). The affected diseases are mainly Parkinson’s disease, systemic lupus erythematosus, and herpes simplex infection, which indicates that microbial dysregulation significantly changes the mechanism of the related conditions. However, whether the microbiota can promote colon cancer needs to be further verified. In addition, the pathways involved in RCC, such as DNA replication, ribosome, aminoacyl-tRNA biosynthesis, and nucleotide excision repair, are more involved in DNA synthesis (Wang et al., 2017). Methane metabolism is closely related to the development of colon cancer (Bertagnolli et al., 1997). The regional comparisons between Xiamen and Harbin, as described above, combined with the species difference analysis yielded results that are consistent with those obtained in previous studies (Wang et al., 2017).

## Conclusions

In summary, our research constitutes the first combined investigation of fecal and tissue samples aiming to explain the pathogenesis of colon cancer in different parts of the colon based on the distribution of microbiota. Based on our data, we determined that the distribution of microbiota in LCC and RCC is significantly distinct and confirmed that the increased (in terms of both type and abundance) pathogenic bacteria found in the left side of the colon are more likely to explain the higher incidence of LCC. In addition, the difference in microbiota between the left- and right-sided colon samples might be instructive for VEGF- and EGFR-targeted therapy. Due to the small sample size used in this study, further research studies based on larger-scale sequencing are necessary. Therefore, the identification of the composition of adherent bacteria in the microbiota at different colon locations is an essential step toward the development of effective prognostic, preventative, or therapeutic strategies.

## Data Availability Statement

The raw reads were deposited into the NCBI Sequence Read Archive (SRA) database. (Accession Number: SRA: SRP258771 and Bioproject PRJNA628032). 

## Ethics Statement

The studies involving human participants were reviewed and approved by Ethic Committee of the First Affiliated Hospital of Harbin Medical University. Written informed consent to participate in this study was provided by the participants’ legal guardian/next of kin.

## Author Contributions

MZ, JZ, and XH designed the study. MZ, JZ, YL, and YX conducted the experiments. ZY, LZ, YZ, LT, and XQ analyzed the results. LZ and YZ collected the clinical samples. MZ, YX, ZY, and JZ wrote the manuscript. LZ and XH edited the manuscript and provided critical comments. All authors contributed to the article and approved the submitted version.

## Funding

This work was primarily supported by the Natural Science Foundation of Fujian Province (No. 2017J01368, No. 2017J01369), the Training Program for Young Talents of Fujian Health System (No. 2016-ZQN-85), the Fujian Provincial Funds for Distinguished Young Scientists (No. 2018D0016) and the Fujian Health Education Joint Research Project (WKJ2016-2-17). 

## Conflict of Interest

The authors declare that the research was conducted in the absence of any commercial or financial relationships that could be construed as a potential conflict of interest. 

## Supplementary Material

Supplementary Figure 1 Beta diversity analysis of different groups in stool samples. R language Vegan software was used for PCoA based on Species. PCoA is based on a distance matrix for dimensionality reduction analysis. Beta diversity indexes (Bray, Jaccard) obtained for the stool samples between the total left and right samples **(A)**, between the left and right samples from Harbin **(B)**, between the left and right samples from Xiamen **(C)**, and between Xiamen and Harbin **(D)**.

Supplementary Figure 2 Beta diversity analysis of different groups in tumor samples. Beta diversity indexes (Bray, Jaccard) obtained for the tumor samples between the total left and right samples **(A)**, between the left and right samples from Harbin **(B)**, between the left and right samples from Xiamen **(C)**, and between Xiamen and Harbin **(D)**.

## Abbreviations

LCC, Left-sided colon cancer; RCC, Right-sided colon cancer; EGFR, Epidermal growth factor receptor; VEGF, Vascular endothelial growth factor; OUT, Operational taxonomic unit.
