# Supplementary figures and images for: Microbial Community Profiling Distinguishes Left-Sided and Right-Sided Colon Cancer
Source: Front Cell Infect Microbiol. 2020 Nov 26;10:498502. doi: 10.3389/fcimb.2020.498502 (PMC7726112; doi:10.3389/fcimb.2020.498502)

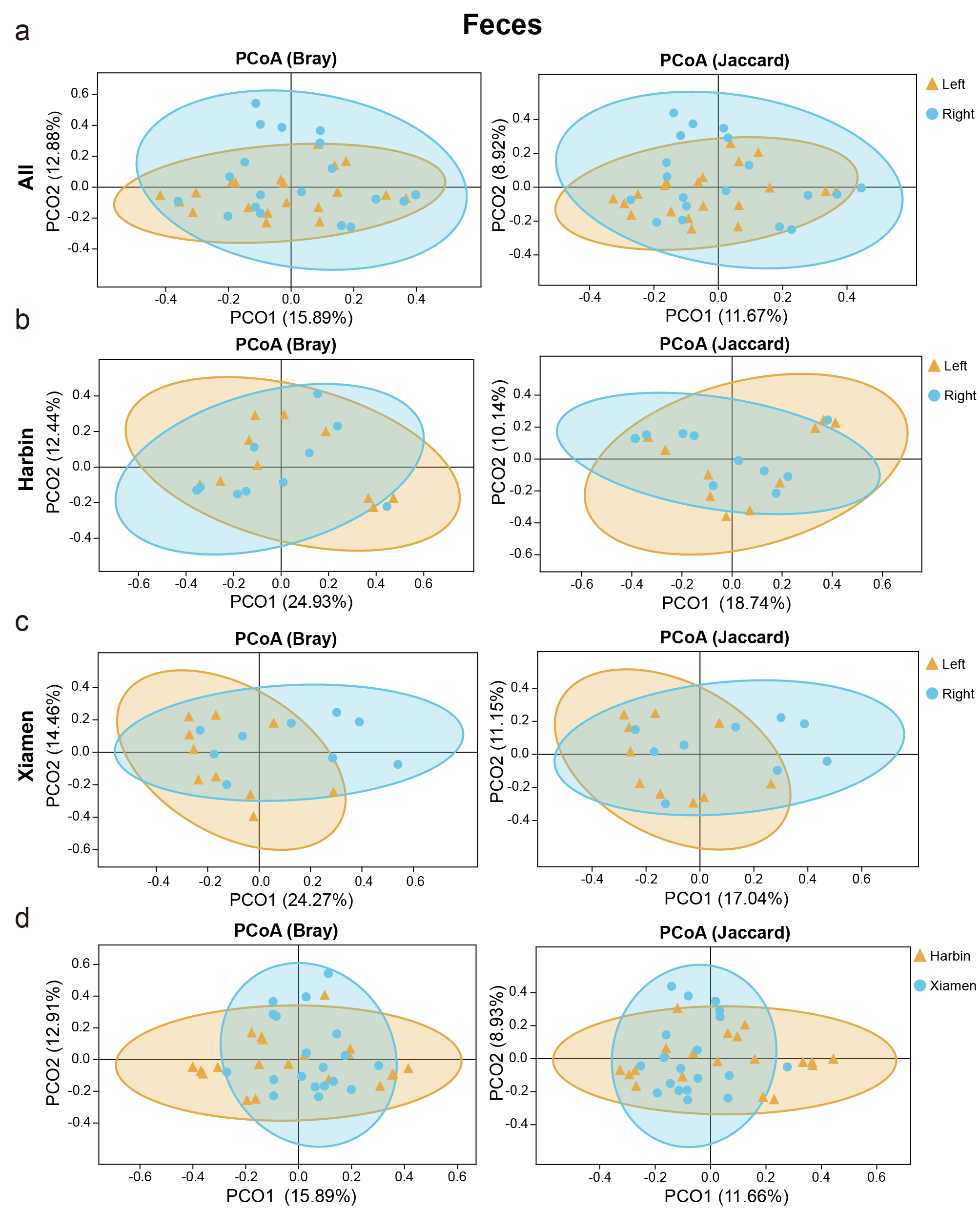

Supplement: Supplementary Figure 1 — Beta diversity analysis of different groups in stool samples. R language Vegan software was used for PCoA based on Species. PCoA is based on a distance matrix for dimensionality reduction analysis. Beta diversity indexes (Bray, Jaccard) obtained for the stool samples between the total left and right samples (A), between the left and right samples from Harbin (B), between the left and right samples from Xiamen (C), and between Xiamen and Harbin (D). [file Image_1.tif]

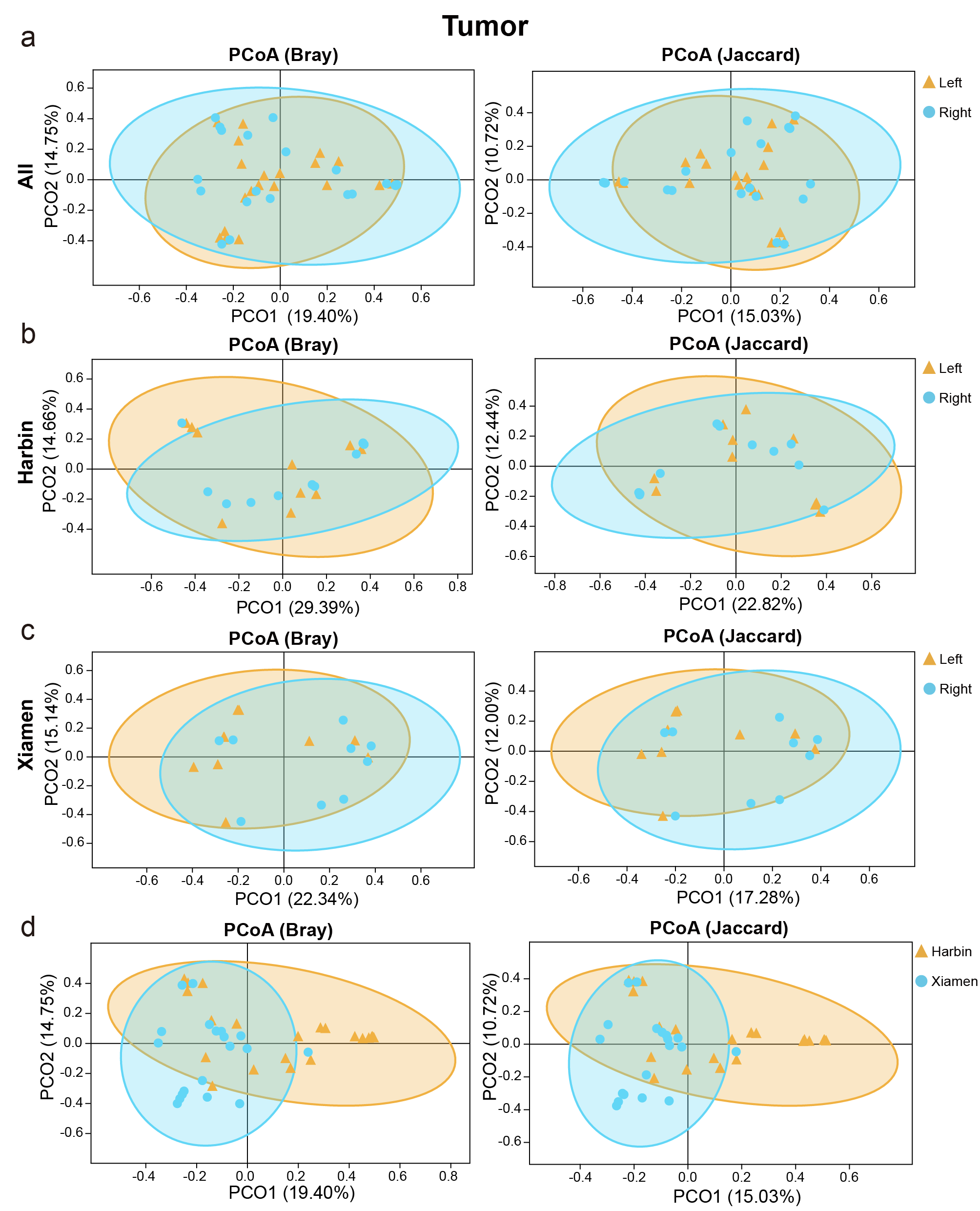

Supplement: Supplementary Figure 2 — Beta diversity analysis of different groups in tumor samples. Beta diversity indexes (Bray, Jaccard) obtained for the tumor samples between the total left and right samples (A), between the left and right samples from Harbin (B), between the left and right samples from Xiamen (C), and between Xiamen and Harbin (D). [file Image_2.tif]
