# Supplementary material for: Microbial Community Profiling Distinguishes Left-Sided and Right-Sided Colon Cancer
Source: Front Cell Infect Microbiol. 2020 Nov 26;10:498502. doi: 10.3389/fcimb.2020.498502 (PMC7726112; doi:10.3389/fcimb.2020.498502)
Supplement: Supplementary file 3 [file DataSheet_1.pdf]

Supplementary Table 1.Differential species between Harbin and Xiamen in the stool samples					
Species	harbin(Mean abundance)	xiamen(Mean abundance)	fold(M-xiamen/M-harbin)	p-value	q-value
Bifidobacterium_animalis	2.81E-04	2.02E-01	7.19E+02	1.58E-08	8.05E-07
Prevotella_buccae_D17	2.67E-03	5.93E-01	2.22E+02	4.93E-08	1.08E-06
Clostridium_baratii	2.79E-03	4.08E-01	1.46E+02	6.33E-08	1.08E-06
Acinetobacter_radioresistens	3.23E-01	1.32E-04	4.08E-04	1.00E-07	1.28E-06
Morganella_morganii_subsp_morganii	1.52E-01	1.63E-04	1.07E-03	2.98E-07	2.15E-06
Clostridium_thiosulfatireducens	0.00E+00	3.00E-02	Inf	3.00E-07	2.15E-06
Odoribacter_sp_Marseille-P2698	1.21E-02	1.43E-01	1.18E+01	3.16E-07	2.15E-06
Acinetobacter_calcoaceticus	1.72E-01	1.11E-04	6.41E-04	3.37E-07	2.15E-06
bacterium_NLAE-zl-G313	4.26E-02	0.00E+00	0.00E+00	3.54E-06	2.01E-05
Prevotella_intermedia	4.64E-02	5.52E-01	1.19E+01	4.52E-06	2.30E-05
Porphyromonas_asaccharolytica	6.91E-02	6.84E-05	9.90E-04	2.13E-05	9.86E-05
Haemophilus_influenzae	0.00E+00	1.25E-02	Inf	2.74E-05	1.16E-04
Shewanella_algae	0.00E+00	1.14E-02	Inf	7.53E-05	2.90E-04
bacterium_endosymbiont_of_Onthophagus_	5.24E-02	7.63E-04	1.46E-02	7.95E-05	2.90E-04
Acinetobacter_johnsonii	2.82E-02	2.16E-04	7.66E-03	1.04E-04	3.52E-04
Collinsella_sp_GD3	0.00E+00	7.45E-03	Inf	4.98E-04	1.59E-03
Bacteroides_eggerthii	5.35E-03	6.71E-02	1.25E+01	6.61E-04	1.98E-03
Bacteroides_coprocola_DSM_17136	5.73E-01	7.49E-01	1.31E+00	9.52E-04	2.70E-03
Collinsella_aerofaciens	3.82E-02	1.50E-01	3.93E+00	1.26E-03	3.23E-03
Clostridium_perfringens	4.99E-01	3.75E-02	7.52E-02	1.27E-03	3.23E-03
Bifidobacterium_dentium	2.60E-02	5.49E-02	2.11E+00	2.28E-03	5.55E-03
Peptoclostridium_difficile	9.88E-02	1.58E-02	1.60E-01	3.72E-03	8.32E-03
Alistipes_sp_AL-1	3.59E-01	1.43E+00	3.99E+00	3.75E-03	8.32E-03
infirmum	4.67E-04	8.94E-03	1.92E+01	5.14E-03	1.09E-02
scindens	8.44E-02	1.63E-02	1.93E-01	7.04E-03	1.44E-02
Megasphaera_micronuciformis	3.23E-03	3.96E-02	1.23E+01	7.51E-03	1.47E-02
Sutterella_wadsworthensis_3_1_45B	2.47E-01	6.41E-02	2.60E-01	7.88E-03	1.48E-02
Bifidobacterium_bifidum	1.15E-02	4.24E-02	3.69E+00	8.13E-03	1.48E-02
Bacteroides_plebeius_DSM_17135	1.98E+00	4.21E-01	2.13E-01	9.00E-03	1.55E-02
Lactobacillus_mucosae	5.05E-02	1.50E-01	2.98E+00	9.12E-03	1.55E-02
Ruminococcus_sp_15975	8.34E-02	1.04E-02	1.25E-01	1.04E-02	1.72E-02

Ruminococcus_sp_UNKMGS-30	5.15E-02	1.60E-01	3.10E+00	1.27E-02	1.96E-02
Parabacteroides_faecis	1.52E-02	2.01E-02	1.32E+00	1.27E-02	1.96E-02
Bacteroides_stercoris_ATCC_43183	2.23E-03	1.11E-02	4.96E+00	2.94E-02	4.40E-02
Clostridium_sp	8.47E-01	2.66E-01	3.14E-01	3.58E-02	5.22E-02
Parabacteroides_distasonis	3.17E+00	8.51E-01	2.69E-01	3.86E-02	5.46E-02
Streptococcus_gallolyticus_subsp_macedoni	5.72E-02	1.09E-01	1.90E+00	4.78E-02	6.58E-02
Lactobacillus_salivarius	6.48E-02	5.17E-02	7.98E-01	1.40E-01	1.88E-01
Streptococcus_anginosus_subsp_anginosus	6.76E-02	4.43E-02	6.55E-01	3.10E-01	4.05E-01
Parabacteroides_goldsteinii	1.16E+00	1.12E-01	9.58E-02	3.76E-01	4.79E-01
Alistipes_sp_N15MGS-157	4.68E-03	1.00E-02	2.14E+00	4.23E-01	5.26E-01
Bacteroides_vulgatus	3.67E-01	4.31E-01	1.17E+00	4.69E-01	5.67E-01
Dialister_pneumosintes	7.40E-02	3.53E-02	4.77E-01	4.81E-01	5.67E-01
Desulfovibrio_desulfuricans	7.46E-03	1.26E-02	1.69E+00	4.89E-01	5.67E-01
Bacteroides_thetaiotaomicron	1.47E+00	9.12E-01	6.19E-01	5.37E-01	6.09E-01
Ruminococcus_sp_N15MGS-57	2.77E-02	4.12E-03	1.49E-01	6.21E-01	6.79E-01
gut_metagenome	6.59E-02	1.11E-01	1.68E+00	6.26E-01	6.79E-01
Bifidobacterium_longum_subsp_longum	3.77E-01	3.77E-01	1.00E+00	7.28E-01	7.63E-01
Bacteroides_uniformis	1.83E+00	1.35E+00	7.38E-01	7.48E-01	7.63E-01
human_gut_metagenome	4.06E-01	1.55E-01	3.82E-01	7.48E-01	7.63E-01
Bacteroides_fragilis	3.41E+00	1.61E+00	4.73E-01	8.51E-01	8.51E-01
