# Supplementary material for: Microbial Community Profiling Distinguishes Left-Sided and Right-Sided Colon Cancer
Source: Front Cell Infect Microbiol. 2020 Nov 26;10:498502. doi: 10.3389/fcimb.2020.498502 (PMC7726112; doi:10.3389/fcimb.2020.498502)
Supplement: Supplementary file 4 [file DataSheet_2.pdf]

Supplementary Table 2. Differential species between Harbin and Xiamen in the tumor samples					
Species	harbin(Mean abundance)	xiamen(Mean abundance)	fold(M-xiamen/M-harbin)	p-value	q-value
Rhizobium_radiobacter	9.62E-04	2.82E-01	2.93E+02	1.12E-07	7.51E-06
Bromus_tectorum	8.57E-05	1.23E-01	1.44E+03	1.79E-07	7.51E-06
Acinetobacter_sp_BFE41A	2.39E-03	5.13E-01	2.15E+02	9.59E-07	2.69E-05
Streptococcus_dysgalactiae_subsp_equisimi	1.41E-02	2.58E+00	1.82E+02	3.54E-06	7.42E-05
Acinetobacter_johnsonii	6.24E-04	8.55E-02	1.37E+02	1.24E-05	1.79E-04
Sphingomonas_paucimobilis	3.10E-04	1.78E-01	5.75E+02	1.28E-05	1.79E-04
Sphingobium_yanoikuyae	7.14E-05	8.12E-02	1.14E+03	2.71E-05	3.25E-04
Clostridium_butyricum	1.66E-03	7.29E-02	4.39E+01	4.20E-05	4.41E-04
Bacteroides_uniformis	2.77E+00	1.96E-01	7.06E-02	6.09E-05	5.69E-04
Parabacteroides_distasonis	3.38E+00	1.66E-01	4.93E-02	7.67E-05	6.44E-04
Prevotella_intermedia	6.58E-02	1.17E+00	1.78E+01	2.20E-04	1.68E-03
Bifidobacterium_bifidum	3.05E-04	1.13E-01	3.71E+02	3.47E-04	2.38E-03
Campylobacter_gracilis	3.76E-04	9.69E-02	2.58E+02	3.69E-04	2.38E-03
Solanum_torvum	1.46E-03	2.15E-02	1.47E+01	5.50E-04	3.30E-03
Flavobacterium_sp_YH1	7.62E-05	2.93E-02	3.84E+02	6.55E-04	3.67E-03
Haemophilus_influenzae	9.14E-04	3.16E-01	3.46E+02	1.14E-03	5.97E-03
Porites_australiensis	6.05E-04	1.98E-01	3.28E+02	1.21E-03	5.99E-03
Parabacteroides_sp_D13	4.09E-02	2.37E-03	5.80E-02	1.40E-03	6.31E-03
Fusobacterium_nucleatum_subsp_animalis	6.38E-04	5.63E-02	8.83E+01	1.43E-03	6.31E-03
Ruminococcus_sp_15975	1.13E-01	9.79E-03	8.66E-02	1.89E-03	7.94E-03
Parabacteroides_merdae	2.65E+00	1.57E-01	5.93E-02	2.13E-03	8.54E-03
Haemophilus_haemolyticus	3.81E-04	6.39E-02	1.68E+02	3.11E-03	1.19E-02
Lactobacillus_murinus	2.38E-04	1.06E-01	4.43E+02	4.18E-03	1.53E-02
Bacteroides_vulgatus	1.33E-01	9.37E-02	7.05E-01	5.48E-03	1.92E-02
Pseudomonas_oryzihabitans	0.00E+00	1.71E-02	Inf	6.44E-03	2.16E-02
Porphyromonas_asaccharolytica	5.77E-02	5.78E-02	1.00E+00	1.14E-02	3.70E-02
Bacteroides_thetaiotaomicron	2.79E+00	1.37E+00	4.92E-01	1.26E-02	3.90E-02
Bifidobacterium_breve	9.81E-04	3.62E-02	3.69E+01	1.33E-02	3.99E-02
Porphyromonas_sp_oral_clone_P4GB_100_	8.38E-04	1.50E-01	1.79E+02	1.46E-02	4.23E-02
Lachnospiraceae_bacterium_615	3.43E-04	7.94E-02	2.32E+02	1.54E-02	4.31E-02
Parabacteroides_goldsteinii	1.43E+00	1.84E-01	1.29E-01	2.39E-02	6.49E-02

Bacillus_smithii	0.00E+00	9.40E-02	Inf	3.09E-02	8.10E-02
Acinetobacter_calcoaceticus	1.05E-01	5.12E-02	4.88E-01	4.54E-02	1.16E-01
Veillonella_parvula	1.91E-02	1.34E-02	7.01E-01	5.05E-02	1.23E-01
bacterium_NLAE-zl-G313	4.88E-02	0.00E+00	0.00E+00	5.11E-02	1.23E-01
Ruminococcus_sp_UNKMGS-30	4.53E-02	9.17E-03	2.02E-01	5.54E-02	1.29E-01
Lactobacillus_aviarius	0.00E+00	5.94E-03	Inf	6.60E-02	1.50E-01
Acinetobacter_radioresistens	1.64E-02	8.42E-05	5.14E-03	8.91E-02	1.97E-01
Sphingomonas_aurantiaca	8.57E-05	1.58E-02	1.84E+02	1.09E-01	2.35E-01
Dialister_pneumosintes	8.16E-02	1.16E-01	1.42E+00	1.18E-01	2.47E-01
unidentified_eubacterium_clone_342	1.34E-02	1.65E-01	1.22E+01	1.24E-01	2.54E-01
aldenense	5.28E-02	7.62E-02	1.44E+00	1.27E-01	2.54E-01
Pseudomonas_pertucinogena	0.00E+00	1.00E-02	Inf	1.42E-01	2.73E-01
Brachyspira_sp_NSH-25	3.69E-03	1.69E-02	4.59E+00	1.44E-01	2.73E-01
Solobacterium_moorei	5.16E-03	1.10E-01	2.12E+01	1.46E-01	2.73E-01
Alistipes_sp_AL-1	4.35E-01	2.97E-01	6.84E-01	1.50E-01	2.74E-01
Bifidobacterium_longum_subsp_longum	2.76E-01	1.57E-01	5.68E-01	1.55E-01	2.76E-01
bacterium_endosymbiont_of_Onthophagus_	1.33E-02	5.84E-03	4.40E-01	1.81E-01	3.17E-01
Streptococcus_anginosus_subsp_anginosus	5.14E-02	2.72E-01	5.30E+00	2.09E-01	3.59E-01
Stenotrophomonas_rhizophila	8.57E-05	6.39E-03	7.46E+01	2.42E-01	4.06E-01
unidentified_rumen_bacterium_12-124	2.71E-04	2.48E-02	9.12E+01	2.75E-01	4.43E-01
leptum	3.43E-04	2.30E-02	6.71E+01	2.75E-01	4.43E-01
Bacteroides_plebeius_DSM_17135	1.87E+00	1.87E+00	1.00E+00	3.10E-01	4.43E-01
Eubacterium_ramulus	8.60E-02	1.39E-01	1.62E+00	3.13E-01	4.43E-01
Treponema_denticola	0.00E+00	1.95E-02	Inf	3.17E-01	4.43E-01
Treponema_succinifaciens_DSM_2489	0.00E+00	1.84E-02	Inf	3.17E-01	4.43E-01
Lactobacillus_harbinensis	0.00E+00	1.22E-02	Inf	3.17E-01	4.43E-01
Paraburkholderia_kururiensis_subsp_kururie	0.00E+00	1.16E-02	Inf	3.17E-01	4.43E-01
delta_proteobacterium_WX152	0.00E+00	9.99E-03	Inf	3.17E-01	4.43E-01
Lactobacillus_agilis	0.00E+00	6.25E-03	Inf	3.17E-01	4.43E-01
Bacteroides_coprocola_DSM_17136	6.21E-01	4.28E-01	6.89E-01	3.35E-01	4.62E-01
Odoribacter_sp_Marseille-P2698	2.28E-02	0.00E+00	0.00E+00	3.68E-01	4.98E-01
Clostridium_sp	7.43E-01	4.03E-01	5.42E-01	4.05E-01	5.40E-01
Bacteroides_fragilis	3.46E+00	4.02E+00	1.16E+00	4.52E-01	5.94E-01
Parabacteroides_faecis	1.69E-02	1.01E-02	5.99E-01	4.99E-01	6.35E-01
Bifidobacterium_dentium	1.70E-02	8.72E-02	5.13E+00	5.03E-01	6.35E-01

Bacillus_coagulans	5.86E-04	1.03E-01	1.75E+02	5.14E-01	6.35E-01
bacterium_YE57	1.00E-04	6.83E-03	6.83E+01	5.14E-01	6.35E-01
human_gut_metagenome	1.39E-02	1.21E-02	8.70E-01	6.20E-01	7.54E-01
Klebsiella_oxytoca	4.53E-03	3.48E-02	7.68E+00	6.35E-01	7.62E-01
Klebsiella_variicola	8.61E-01	2.61E-01	3.04E-01	7.68E-01	9.09E-01
Clostridium_perfringens	3.31E-01	2.26E-01	6.83E-01	7.96E-01	9.28E-01
gut_metagenome	4.40E-02	5.21E-01	1.18E+01	8.25E-01	9.49E-01
Streptococcus_gallolyticus_subsp_macedon[illegible]	3.10E-02	2.28E-01	7.33E+00	8.38E-01	9.51E-01
Lactobacillus_fermentum	7.43E-04	1.61E-02	2.16E+01	8.50E-01	9.53E-01
Lactobacillus_mucosae	2.05E-02	3.43E-02	1.67E+00	9.01E-01	9.57E-01
Bacteroides_eggerthii	6.19E-03	2.93E-02	4.74E+00	9.11E-01	9.57E-01
Clostridium_baratii	3.13E-03	2.44E-02	7.81E+00	9.23E-01	9.57E-01
Bifidobacterium_adolescentis	9.70E-03	4.00E-03	4.13E-01	9.31E-01	9.57E-01
Streptococcus_mutans	7.57E-04	1.09E-02	1.44E+01	9.38E-01	9.57E-01
mouse_gut_metagenome	1.38E-04	1.34E-02	9.70E+01	9.43E-01	9.57E-01
Collinsella_sp_GD3	7.62E-05	5.64E-03	7.41E+01	9.43E-01	9.57E-01
scindens	8.01E-02	1.55E-01	1.94E+00	9.46E-01	9.57E-01
Lactobacillus_salivarius	2.30E-02	4.09E-02	1.78E+00	9.76E-01	9.76E-01
