# Supplementary material for: Microbial Community Profiling Distinguishes Left-Sided and Right-Sided Colon Cancer
Source: Front Cell Infect Microbiol. 2020 Nov 26;10:498502. doi: 10.3389/fcimb.2020.498502 (PMC7726112; doi:10.3389/fcimb.2020.498502)
Supplement: Supplementary file 5 [file DataSheet_3.pdf]

Supplementary Table 3. Alpha diversity index in Xiamen and Harbin.

Group	alpha diversity index	P value
Harbin-VS-Xiamen(feces)	Chao1	1.24E-04
	Ace	7.20E-05
	Sobs	8.68E-04
Harbin-VS-Xiamen(tumor)	Chao1	3.36E-02
	Ace	2.17E-02
